# Luminal A Breast Cancer Co-expression Network: Structural and Functional Alterations

**DOI:** 10.3389/fgene.2021.629475

**Published:** 2021-04-20

**Authors:** Diana García-Cortés, Enrique Hernández-Lemus, Jesús Espinal-Enríquez

**Affiliations:** ^1^Computational Genomics Division, National Institute of Genomic Medicine, Mexico City, Mexico; ^2^Programa de Doctorado en Ciencias Biomédicas, Universidad Nacional Autónoma de México, Mexico City, Mexico; ^3^Centro de Ciencias de la Complejidad, Universidad Nacional Autónoma de México, Mexico City, Mexico

**Keywords:** loss of long range co-expression, gene co-expression networks, Luminal A breast cancer, breast cancer, transcription factor analysis, CTCF binding site analysis

## Abstract

Luminal A is the most common breast cancer molecular subtype in women worldwide. These tumors have characteristic yet heterogeneous alterations at the genomic and transcriptomic level. Gene co-expression networks (GCNs) have contributed to better characterize the cancerous phenotype. We have previously shown an imbalance in the proportion of intra-chromosomal (*cis-*) over inter-chromosomal (*trans-*) interactions when comparing cancer and healthy tissue GCNs. In particular, for breast cancer molecular subtypes (Luminal A included), the majority of high co-expression interactions connect gene-pairs in the same chromosome, a phenomenon that we have called loss of *trans-* co-expression. Despite this phenomenon has been described, the functional implication of this specific network topology has not been studied yet. To understand the biological role that communities of co-expressed genes may have, we constructed GCNs for healthy and Luminal A phenotypes. Network modules were obtained based on their connectivity patterns and they were classified according to their chromosomal homophily (proportion of *cis-/trans-* interactions). A functional overrepresentation analysis was performed on communities in both networks to observe the significantly enriched processes for each community. We also investigated possible mechanisms for which the loss of *trans-* co-expression emerges in cancer GCN. To this end we evaluated transcription factor binding sites, CTCF binding sites, differential gene expression and copy number alterations (CNAs) in the cancer GCN. We found that *trans-* communities in Luminal A present more significantly enriched categories than *cis-* ones. Processes, such as angiogenesis, cell proliferation, or cell adhesion were found in *trans-* modules. The differential expression analysis showed that FOXM1, CENPA, and CIITA transcription factors, exert a major regulatory role on their communities by regulating expression of their target genes in other chromosomes. Finally, identification of CNAs, displayed a high enrichment of deletion peaks in *cis-* communities. With this approach, we demonstrate that network topology determine, to at certain extent, the function in Luminal A breast cancer network. Furthermore, several mechanisms seem to be acting together to avoid *trans-* co-expression. Since this phenomenon has been observed in other cancer tissues, a remaining question is whether the loss of long distance co-expression is a novel hallmark of cancer.

## 1. Background

Gene co-expression networks (GCN) enable the study of interactions of highly correlated genes in a transcriptional program, capturing global and local connectivity properties emerging from those interactions (Sonawane et al., 2019). These type of networks are built from gene expression profiles, a measurable output of transcription. Therefore, they outline the contribution of the regulatory elements operating at different levels of the transcription process to ensure the expression of specific sets of genes. In this sense, GCNs might provide insights about shared regulatory mechanisms and their alterations in a disease, such as cancer (Emmert-Streib et al., 2014; Yang et al., 2014; Wu et al., 2019; Liao et al., 2020). Those alterations in cancer disrupt the transcriptional process and lead to altered gene expression and the promotion of tumor progression (Garraway and Lander, 2013; Lee and Young, 2013).

There are multiple studies where GCNs are constructed and important aspects of the connectivity structure are analyzed to identify genes prognosis markers (Hsu et al., 2019), metabolic deregulation (Serrano-Carbajal et al., 2020), and differences in transcriptional profiles (van Dam et al., 2018).

In breast cancer GCNs, there is an imbalance in the proportion of intra-chromosomal (*cis-*) over inter-chromosomal (*trans-*) gene co-expression interactions, meaning that the majority of high co-expression links connect gene-pairs in the same chromosome (Espinal-Enríquez et al., 2017; de Anda-Jáuregui et al., 2019a; Dorantes-Gilardi et al., 2020). This phenomenon has been called loss of long distance co-expression. Furthermore, a highly localized co-expression pattern associated with chromosome cytobands has been observed (García-Cortés et al., 2020). These features are not present in the healthy tissue GCN. In the entire set of co-expression interactions, the loss of long distance co-expression in breast cancer (measured in base pairs) subtypes is displayed as a decay in the *cis-* co-expression values dependent on gene physical distance (de Anda-Jáuregui et al., 2019b; García-Cortés et al., 2020).

The structural characteristics evaluated in the co-expression networks are different for each breast cancer molecular subtype, displaying another instance of their emblematic heterogeneity (Alcalá-Corona et al., 2017, 2018a). The four breast cancer molecular subtypes, Luminal A, Luminal B, HER2+ and Basal-like, are classified according to their gene expression profiles and they represent different cancer manifestations, with distinct molecular traits, genomic alterations, and prognosis (Perou et al., 2000; Prat and Perou, 2011; Berger et al., 2018). Hormone status, evaluated through the expression of estrogen and progesterone receptors (ER and PR correspondingly), and the presence of human epidermal growth factor receptor 2 (HER2), play a major role for breast cancer molecular subtypes characterization and the election of therapeutic strategies (Zhang et al., 2014).

Luminal A is the most frequent breast cancer molecular subtype. Almost a half of the total cases of breast cancer correspond to this phenotype (Fan et al., 2006). These tumors are often positive to estrogen receptor (ER) and negative to ERBB2 receptor, and they also present overexpression on the ER-regulated genes. This subtype is associated with highest median survival, best prognosis (Hu et al., 2006), and lower recurrence rates (Arvold et al., 2011; Metzger-Filho et al., 2013).

Nevertheless, clinical and molecular heterogeneity is present within Luminal A tumors, where differences in genomic alterations have been potentially associated with resistance to endocrine therapy (Ciriello et al., 2013).

Additionally, the Luminal A GCN presents the least dissimilar structure compared with the healthy GCN (García-Cortés et al., 2020). A relevant measure to analyze differences in cancer GCNs, is the size of connected components. In the case of healthy GCN, as well as in the case of Luminal A GCN, they present a giant component (a set of connected genes that contains more than the half of the total amount of nodes in the networks). The other breast cancer subtype GCNs have only small intra-chromosomal connected components. Furthermore, Luminal A GCN is the one with the highest number of inter-chromosomal (*trans-*) interactions.

The structure of a GNC is often organized into *communities* or modules (Alcalá-Corona et al., 2016), this is, subsets of connected genes so that the density of within-connections is higher than that of between-connections (Girvan and Newman, 2002; Porter et al., 2009; Fortunato and Hric, 2016; Alcalá-Corona et al., 2018a). In the case of GCNs, communities may correspond to a co-regulated set of genes (Wilkinson and Huberman, 2004; Zhu et al., 2008; Cantini et al., 2015). The structure of said modules may capture the phenomenology behind biological processes (Alcalá-Corona et al., 2017, 2018a,b).

Being the subtype with the best prognosis, the most similar co-expression network, and taking into account that community structure in GCN may be implicated in the functional regulation of a cancerous phenotype, in this work we analyzed the structure of communities of the Luminal A GCN, in order to determine the relevance of the loss of long distance co-expression in the biological functions associated to that network. Additionally, we evaluated possible mechanisms for which we observe the preference for *cis-* interactions in this breast cancer subtype. We analyzed the influence of differential gene expression, transcription factor binding sites, copy number alterations, and CTCF binding sites, in order to understand the regulatory mechanisms underlying the appearance of the loss of long distance interactions in cancer GCNs.

## 2. Results and Discussion

### 2.1. Community Structure Displays Loss of *trans-* Co-expression

**Figure 1:**
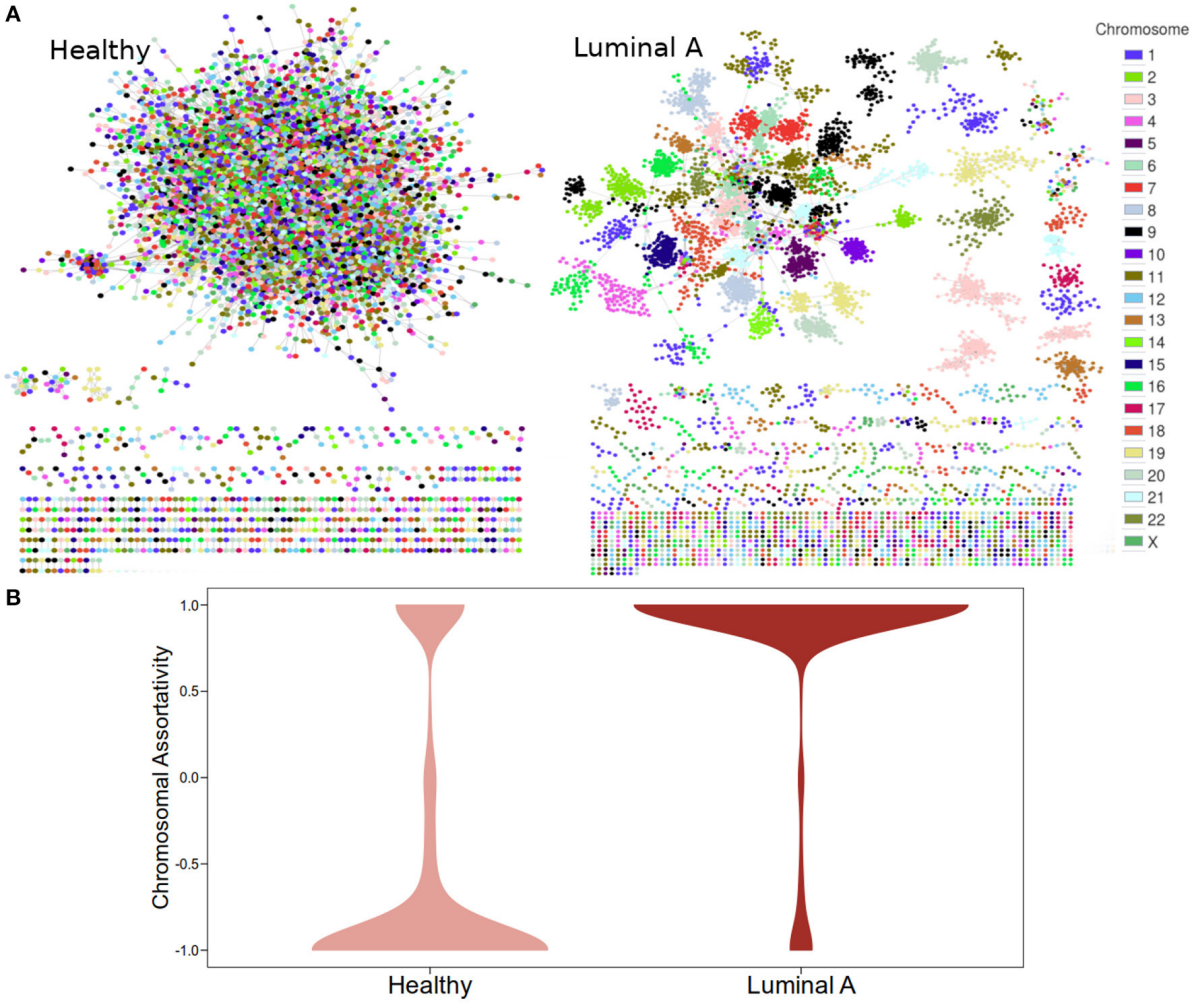
Co-expression networks for Healthy and Luminal A tissue. **(A)** GCNs built from the 20,217 most significant gene pair mutual information values for both phenotypes. Node colors are assigned according to the chromosome where each gene is located. **(B)** Distribution of chromosomal assortativity in network communities.

Figure 1A displays GCNs built from the 20,217 (see Methods section) most significant mutual information interactions in the Luminal A and the Healthy co-expression profiles. Genes are colored according to the chromosome where they are located. As previously reported, the Healthy GCN has a giant component with interactions linking genes from different chromosomes. The Luminal A network also has a giant component but the layout suggests that genes from the same chromosome are preferentially linked.

To evaluate the previous observation, communities were detected in both networks using four algorithms for weighted networks implemented in the igraph package: Fast Greedy, Infomap, Leading Eigenvector, and Louvain. Supplementary Material 1 presents results for all algorithms. Jaccard indexes where calculated among communities detected by the four algorithms. More than 95% of the total number of communities detected by Fast Greedy, Leading Eigenvector, and Louvain have a Jaccard Index equal to 1, while Infomap displays more dissimilar results. Given that Louvain presents the highest modularity values, results for this algorithm are presented in the main text. Table 1 contains the number of communities and modularity values for the four algorithms applied to the Healthy and the Luminal A network.

**Table 1:** Features of *cis*- and *trans*- chromosomal communities in the Luminal A and the Healthy gene co-expression network.

**Algorithm**	**Healthy**							**Luminal A**						
	**Modularity**	**Communities**		**Size** **≥** **5**		**Enriched communities**		**Modularity**	**Communities**		**Size** **≥** **5**		**Enriched communities**	
		***cis-***	***trans-***	***cis-***	***trans-***	***cis-***	***trans-***		***cis-***	***trans-***	***cis-***	***trans-***	***cis-***	***trans-***
Fast Greedy	0.703	75	325	0	50	0	14	0.934	614	87	77	40	9	20
Infomap	0.674	83	768	1	386	1	47	0.907	826	93	194	39	16	20
Leading Eigenvector	0.696	71	283	1	32	1	18	0.892	594	84	58	37	9	20
Louvain	0.752	71	291	0	41	0	17	0.935	614	87	77	40	9	20

Chromosomal assortativity, *ASS*_*chr*_ was calculated by taking the number of intra-chromosomal links minus the number of inter-chromosomal links divided by the total number of links in a community. Figure 1B displays the distribution of the *ASS*_*chr*_ in both networks in the form of violin plots. The differences in the distributions allow us to confirm the loss of *trans-* interactions in the Luminal A GCN.

### 2.2. Specific *trans-* Communities in the Luminal A GCN Are Highly Associated With Biological Processes

To identify the functional role of the highly co-expressed groups of genes identified by network communities, an overrepresentation analysis was performed, using the biological process category in Gene Ontology (GO). Results for all algorithms are presented in Table 1. *-cis* communities are the ones having *ASS*_*chr*_ equals to 1.

**Figure 2:**
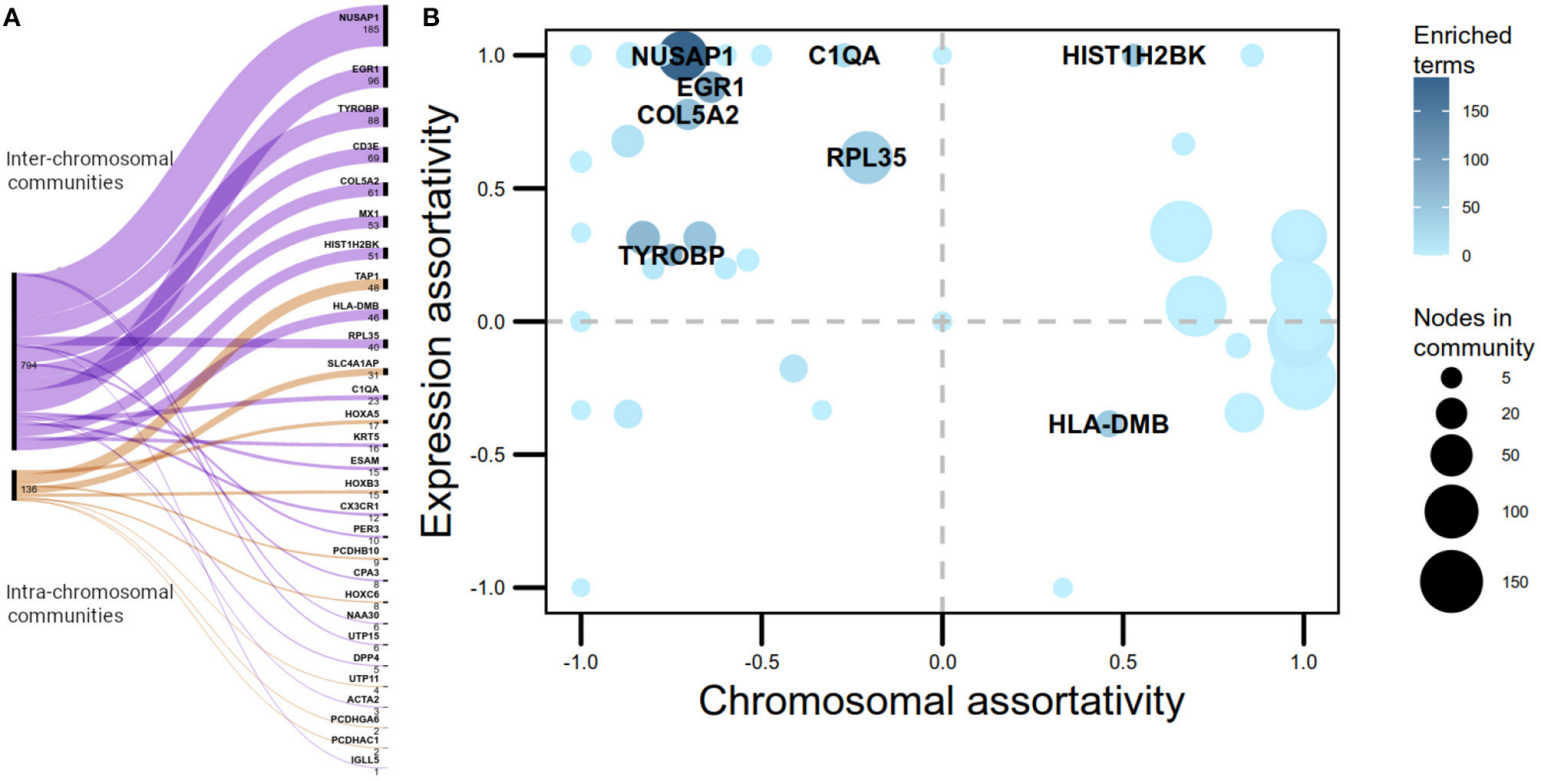
*cis-* and *trans-* communities in the Luminal A network. **(A)** Alluvial graph displaying the proportion of overrepresented Gene Ontology biological processes per community in *trans-* (purple) and *cis-* (orange) communities. The name assigned for each community is the name of the gene with highest page rank value. **(B)** Communities plotted according to their chromosomal and expression assortativity values. Dot sizes correspond to the number of nodes in the community and node color, to the number of overrepresented GO terms. Communities with more than 20 terms are highlighted. Notice that the quadrant with more enriched communities is the one with high expression assortativity and low chromosomal assortativity.

Half of the *-trans* communities with more than five nodes extracted by the Louvain algorithm in the Luminal A GCN were associated with biological processes. However, only 12% of the *-cis* communities where enriched. Despite having a larger number of intra-chromosomal *cis-* communities in the Luminal A network, the majority of communities with statistically significant biological processes associated are *trans-*. Figure 2A presents a visual representation in the form of an alluvial plot. There, the width of each line corresponds to the number of significantly enriched processes for a given community, named by the gene with highest page rank centrality. The difference in the amount of *cis-* and *trans-* communities with associated functions, may reflect that the set of biological processes annotated in GO do not tend to exhibit a bias toward an specific chromosome contrary to what it is observed in the Luminal A GCN communities.

There is a wide variety in the biological enriched processes in the Luminal A *trans-* communities. Processes associated with regulation of transcription, telomere maintenance, and regulation of cell division as well as gene silencing are found. Supplementary Table 1 contains the entire set of significantly overrepresented processes for Luminal A and healthy GCNs, as well as the shared enriched terms between both networks.

On the other hand, the enriched Luminal A *cis-* communities are mainly composed of gene families located at the same regions in the genome. In this group we have the HOXA, HOXB, and HOXC genes, which are important for embryogenesis. They have been found to be expressed in normal and neoplastic breast tissue (Cantile et al., 2003), with altered patterns of expression levels in breast cancer molecular subtypes. In particular, HOXA genes in Luminal A subtype, have shown underexpression associated with the acquisition of repressive epigenetic marks, such as hypermethylation (Novak et al., 2006; Kamalakaran et al., 2011; Hur et al., 2014).

Protocadherins (PCDHA, PCDHB, and PCDHG genes) were also identified as three distinct *cis-* communities in the Luminal A network. Protocadherin genes were previously identified as the most densely connected component (almost a clique) in a breast cancer network (Espinal-Enríquez et al., 2017). There, it was also shown that all protocadherins resulted underexpressed. The observed underexpression of this cluster coincides with a reported hypermethylation of protocadherins in breast cancer (Novak et al., 2008).

In the Healthy network 41% of the *trans-* communities were associated with biological processes, and no *cis-* communities where enriched due to the fact that *cis-* communities identified in this network have <5 genes (the threshold set for the overrepresentation analysis, see Methods). The set of terms includes mostly metabolism-associated process, cell division, and mitochondrial functions.

The Healthy and the Luminal A GCN share 24 communities of only two nodes. Additionally, there is one community named HLA-DRB1 in the Healthy GCN, and HLA-DMB in the Luminal A GCN, with a Jaccard Index of 0.916. This community is associated with activation of the immune response, and it is composed by MHC class II HLA genes located on chromosome 6 region p21.32, plus CIITA (Class II Major Histocompatibility Complex Transactivator), on Chromosome 16, and CD74, located on chromosome 5, only in the Luminal A community.

One pair of communities named CPA3 in both networks share the set of associated processes, but displays a Jaccard index of 0.705 regarding their gene sets. Processes include peptide hormone processing and regulation of systemic arterial blood pressure. Members of this community, such as TPSAB1, CMA1, CTSG, CPA3, HDC, and MS4A2, are commonly found in Mast Cells expression, part of the immune response and usually recruited to breast tumors (Aponte-López et al., 2020). The presence of these immune-system associated communities as high co-expression sets in both networks might be an instance of multiple cell types present in the sample.

### 2.3. *trans-* Communities in the Luminal A Network Present Different Patterns of Differential Expression

Once we observed that biological processes were significantly associated with *trans-* communities, a differential expression analysis was performed to assess the influence of altered gene expression in *trans-* communities and their processes. Supplementary Figure 1 presents the differential expression representation in the GCN and Supplementary Table 2 contains the log2 fold change (LFC) values for each gene in the network.

The number of links joining genes with the same sign of LFC, minus the number of links between genes with different sign of LFC, over the total number of links, was computed per community as a measure of differential gene expression assortativity (*ASS*_*dge*_). Figure 2B plots *ASS*_*dge*_ and *ASS*_*chr*_ for *trans-* communities, as well as the number of associated GO terms. Highly enriched communities (>20 GO terms) are highlighted. The majority of these communities are placed in the first quadrant of the plot, meaning that their genes tend to have similar differential expression but they are placed in different chromosomes. Moreover, those communities are not in the top-10 regarding size, hence functional association in *-trans* communities appears to be influenced by high *ASS*_*dge*_ and low *ASS*_*chr*_ values.

**Figure 3:**
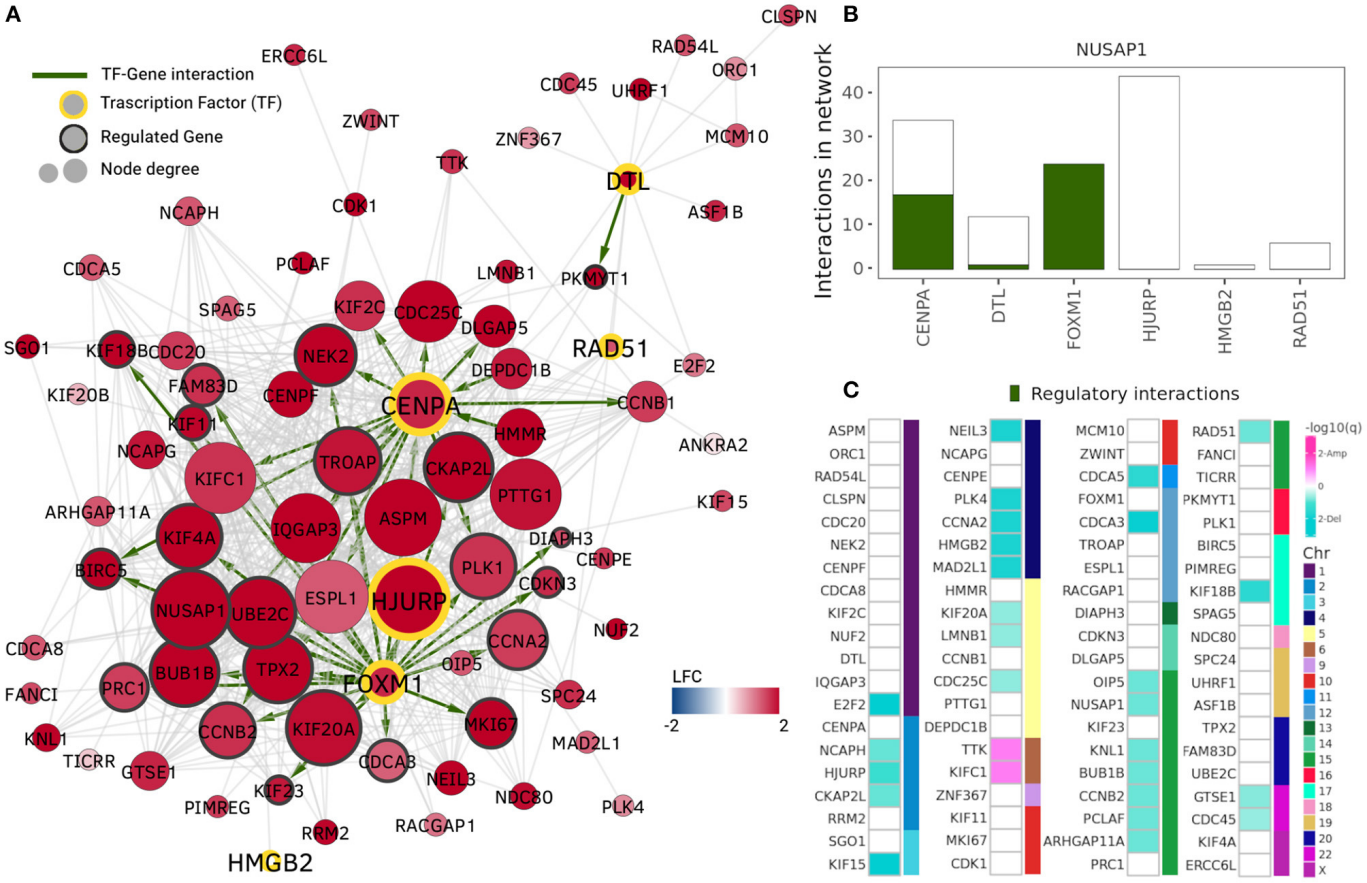
NUSAP1 community. The NUSAP1 community is the community with the highest number of enriched terms and highest expression assortativity. **(A)** Nodes and edges in the NUSAP1 community. Node colors represent log2 fold change, thus, the entire community is overexpressed. Transcription factors (TF) are highlighted by a yellow border and their regulated genes (genes with at least one TF binding site in GTRD) are identified by a gray border. Green edges indicate regulatory interactions. **(B)** Transcription factors in the NUSAP1 community. Total number of network interactions for each TF. The number of interactions identified as regulatory is displayed in green. **(C)** Copy number alteration peaks in the NUSAP1 community. Squares represent each gene in the community and they are ordered according to the chromosome where they are located. Turquoise squares depict genes in deletion peaks while pink ones represent amplification events.

The community with the highest number of enriched GO terms is the NUSAP1 community which also contains highly overexpressed genes only (Figure 3A). Its enriched terms are associated with nuclear division, DNA replication, chromatid segregation, and cell cycle checkpoints, i.e., cell division processes. This community shares a Jaccard index of 0.5 regarding gene members and 0.718 regarding GO associated terms with the MKI67 community in the Healthy network.

NUSAP1 has already been identified as a hub gene in a network of ER positive breast cancer tumor tissues of patients treated with tamoxifen, and derived from a similar methodology but using micro-array data (Liu et al., 2015). In that study, five hub genes with high expression levels strongly associated with poor survival were identified, and four of them: CDK1, DLGAP5, NUSAP1 and RRM2, belong to this particular community.

High expression of several genes in this community, including NUSAP1, was also observed in patients with Luminal A breast cancer and obesity (Nuncia-Cantarero et al., 2018). Nuncia-Cantarero et al. reported 39 genes related with a poor outcome group for patients with both conditions and 26 are found in this community, including FOXM1 (Forkhead box proteinM1), a transcription factor that has been identified as a potential therapeutic target for breast cancer (Lu et al., 2018), highly associated with luminal tumors and ER expression (Millour et al., 2010; Carr et al., 2012).

Table 2 shows the 39 genes reported in Nuncia-Cantarero et al. (2018). The coincident genes found in our network community are bold and their corresponding log2 fold change values are displayed. Interestingly, none of the genes presented in Nuncia-Cantarero et al. (2018) are in the Luminal A GCN but those found in the NUSAP1 community.

**Table 2:** Previously reported genes in the NUSAP1 community.

**Gene**	**Gene name**	**LFC**
**NEK2**	**Serine/threonine-protein kinase Nek2**	3.564
**KIF4A**	**Kinesin Family Member 4**	3.098
**ASPM**	**Abnormal spindle-like microcephaly-associated protein**	2.567
**CENPF**	**Centromere protein F**	2.567
**TPX2**	**Protein TPX2**	2.567
**KIF18B**	**Kinesin Family Member 18B**	2.396
**CDC25C**	**M-phase inducer phosphatase**	2.316
**DLGAP5**	**Disks large-associated protein 5**	2.297
**NUSAP1**	**Nucleolar and spindle-associated protein**	2.223
**MKI67**	**Proliferation marker protein Ki-67**	2.191
**UBE2C**	**Ubiquitin-conjugating enzyme E2**	2.173
**HMMR**	**Hyaluronan mediated motility receptor**	2.162
**BUB1B**	**Mitotic checkpoint serine/threonine-protein kinase**	2.157
**BIRC5**	**Baculoviral IAP repeat-containing protein**	2.057
**CDK1**	**Cyclin-dependent kinase**	2.012
**KIF11**	**Kinesin Family Member 11**	1.963
**RRM2**	**Ribonucleoside-diphosphate reductase subunit M2**	1.961
**KIF20A**	**Kinesin Family Member 20**	1.898
**ISG15**	**Ubiquitin-like protein ISG15**	1.789
**GTSE1**	**G2 and S phase-expressed protein**	1.714
**FOXM1**	**Forkhead box protein M1**	1.699
**CCNB2**	**G2/mitotic-specific cyclin-B2**	1.621
**CCNB1**	**G2/mitotic-specific cyclin-B**	1.523
**PRC1**	**Protein regulator of cytokinesis**	1.504
**KIF15**	**Kinesin Family Member 15**	1.425
**ZWINT**	**ZW10 interactor**	1.416
**OIP5**	**Protein Mis18-beta**	1.299
BUB1	Mitotic checkpoint serine/threonine-protein kinase BUB1	
CEP55	Centrosomal protein of 55 kDa	
EZH2	Histone-lysine N-methyltransferase EZH2	
GDP-15	Growth/differentiation factor 15	
KIAA0101	PCNA-associated factor	
MELK	Maternal embryonic leucine zipper kinase	
MMP1	Matrix Metallopeptidase	
MYBL1	MYB Proto-Oncogene Like	
PBK	PDZ Binding Kinase	
RIPPLY3	Protein ripply3	
TOP2A	DNA topoisomerase 2-alpha	
TYMS	Thymidylate synthase	

*39 Genes reported in Nuncia-Cantarero et al. (2018), related with poor outcome group for patients with obesity and Luminal A breast cancer. Highlighted genes are present in the NUSAP1 community. Their corresponding log2 fold change value is also displayed. Notice that all concordant genes are overexpressed*.

From the highly enriched communities, RPL35 is the one with more genes. The majority of them are ribosomal proteins; therefore, among the enriched GO terms we find ribosome biogenesis, large and small ribosomal subunit assembly, as well as regulation of ubiquitin-protein transferase activity. Riboproteins in this community are mostly underexpressed (Supplementary Figure 2). Low levels of expression have been reported in breast cancer for RPL5 and RPL11, associated with a mechanism of apoptosis inhibition through P53 degradation (Tong et al., 2020), and induction of proliferation in MCF7 cells, a Luminal A-derived cell type (Fancello et al., 2017). It has been shown that riboproteins have high co-expression values in other gene co-expression networks (Prieto et al., 2008; Wang et al., 2020a,b). The finding of highly co-expressed cluster of riboproteins reported here, reinforces the fact that these GCNs are coherent and represent with some accuracy the actual co-expression landscape in Luminal A breast cancer.

To our knowledge, coordinated underexpression of ribosomal genes in a breast cancer subtype has not previously been described. On the contrary, an increased ribosomal content has been recently found to contribute to proliferative and metastatic potential in breast cancer circulating tumor cells (Ebright et al., 2020). This discrepancy may be due to the fact that the overexpression of RPL transcripts, such as RPL15 observed in Ebright et al. (2020), was reported for circulating tumor cells. These tumor cells present additional alterations in their transcriptional profile, and they have acquired a highly proliferative capacity. Hence, the underexpression of ribosomal genes in the Luminal A network may be an indicative that the tumors are not as invasive as other subtypes. It is worth noticing again that Luminal A breast cancer subtype is the less aggressive, the one with the best prognostic and also the best in terms of response to therapy.

### 2.4. Effects of Transcription Factors and CNAs in *trans-* Communities

The general overexpression trend observed in the NUSAP1 community, and underexpression in the RPL35 module, suggested a contribution of altered mechanisms of transcriptional regulation promoting the formation of high co-expression clusters. To evaluate this, we analyzed the contribution of regulatory interactions from transcription factors (TFs) and the presence of deletion and amplification peaks in the Luminal A network communities.

TFs in the ten highlighted communities from Figure 2B were identified using data from the Gene Transcription Regulation Database (GTRD) (Yevshin et al., 2018). Five communities included at least one gene reported as TF in GTRD. The total number of interactions for these genes in the NUSAP1 community is presented in Figure 3B, where the number of genes having at least one binding site in the promoter region (1,000 bp upstream, 100 bp downstream from starting point) is shown in green. It can be observed that FOXM1 transcription factor has its entire set of adjacent links marked as regulatory interactions.

As stated in the previous section, the NUSAP1 community contains interactions that have been reported in luminal associated breast cancer phenotypes. Particularly, the FOXM1 transcriptional network was identified as the largest regulon by GPU-ARACNE, the accelerated parallel implementation of ARACNE, the algorithm used here to infer the gene co-expression networks (He et al., 2017). He et al. identified 121 FOXM1 interactions with 14 experimentally validated targets.

In the NUSAP1 community, FOXM1 has 24 co-expression interactions with other genes in the module. All of these interacting genes contain a FOXM1 binding site in their promoter region according to the data gather by GTRD. From these 24 regulated genes, eight intersect with the experimentally validated targets reported in He et al. (2017).

Centromere protein A or CENPA, is another important transcription factor with overexpression in the NUSAP1 community. It regulates centromere integrity and chromosome segregation. This TF was identified in a mRNA signature correlated with lower survival ratio in Luminal A breast cancer (Xiao et al., 2018). One of its interacting proteins, HJURP, required for CENPA centromeric localization, is also a member of this community. HJURP mRNA expression level has been significantly associated with estrogen and progesterone receptor, and reported as clinically relevant for Luminal A breast cancer patients (Hu et al., 2010; Montes de Oca et al., 2015). Although HJURP is the transcription factor with more adjacent links in the NUSAP1 community, none of them was identified as a regulatory interaction; instead, HJURP was identified as regulated by FOXM1.

The remaining overexpressed TFs in the NUSAP1 community have also been found to play a role in the luminal breast cancer phenotype. Increased mRNA expression of RAD51, a gene in the double-strand breaks repair pathway, is associated with higher risk of tumor relapse and distant metastases in estrogen receptor positive breast cancer tumors (Barbano et al., 2011; Nieto-Jiménez et al., 2017). Overexpression of DTL and HMGB2 has also been associated with tumor progression in breast cancer (Perez-Peña et al., 2017; Fu et al., 2018), and resistance to endocrine therapies (Redmond et al., 2015). These results suggest a strong contribution of TFs, particularly from FOXM1 and CENPA, and their interactions found in the NUSAP1 community, to the process of tumorigenesis and progression in Luminal A breast cancer.

Gene copy number alteration (CNA) is a common trait of genomic instability in cancer and their presence has therapeutic relevance in breast cancer, specially for the Her2 enriched subtype (Andre et al., 2009; Inaki et al., 2014). Different levels of correlation have been identified between DNA amplification and deletion events, mRNA, and protein expression values in breast cancer, (Myhre et al., 2013), showing that it is not an homogeneous mechanism of altered expression. However, given the possible effect and importance for the breast cancer phenotype, amplification and deletion peaks may play a role in the formation of high co-expression clusters in the Luminal A network. For instance, in the case of breast cancer, correlation between CNVs and gene expression could reach until 25% (Lachmann, 2016).

Those gene expression alterations may influence importantly in the co-expression landscape. In Lachmann (2016), it was reported that CNVs may impact importantly the co-expression program, in particular for transcription factor targets.

To evaluate the role of CNVs in the Luminal A GCN, we obtained amplification and deletion peaks using the GISTIC2 algorithm (Mermel et al., 2011). Figure 3C presents the results for the NUSAP1 community. Turquoise squares represent genes in which a deletion has been observed, meanwhile amplifications are depicted in pink squares. Since the NUSAP1 community is *trans-*, the chromosome in which those genes are located is also depicted.

As observed, the majority of genes with copy number alterations correspond to deletions. Only two genes, TTK and KIFC1 (Chr6) present amplifications. However, 52 out of 80 genes do not present changes in copy number. This result shows that, at least for the NUSAP1 community, which is the one with the most differentially expressed genes, CNAs do not significantly influence neither expression nor co-expression patterns.

**Figure 4:**
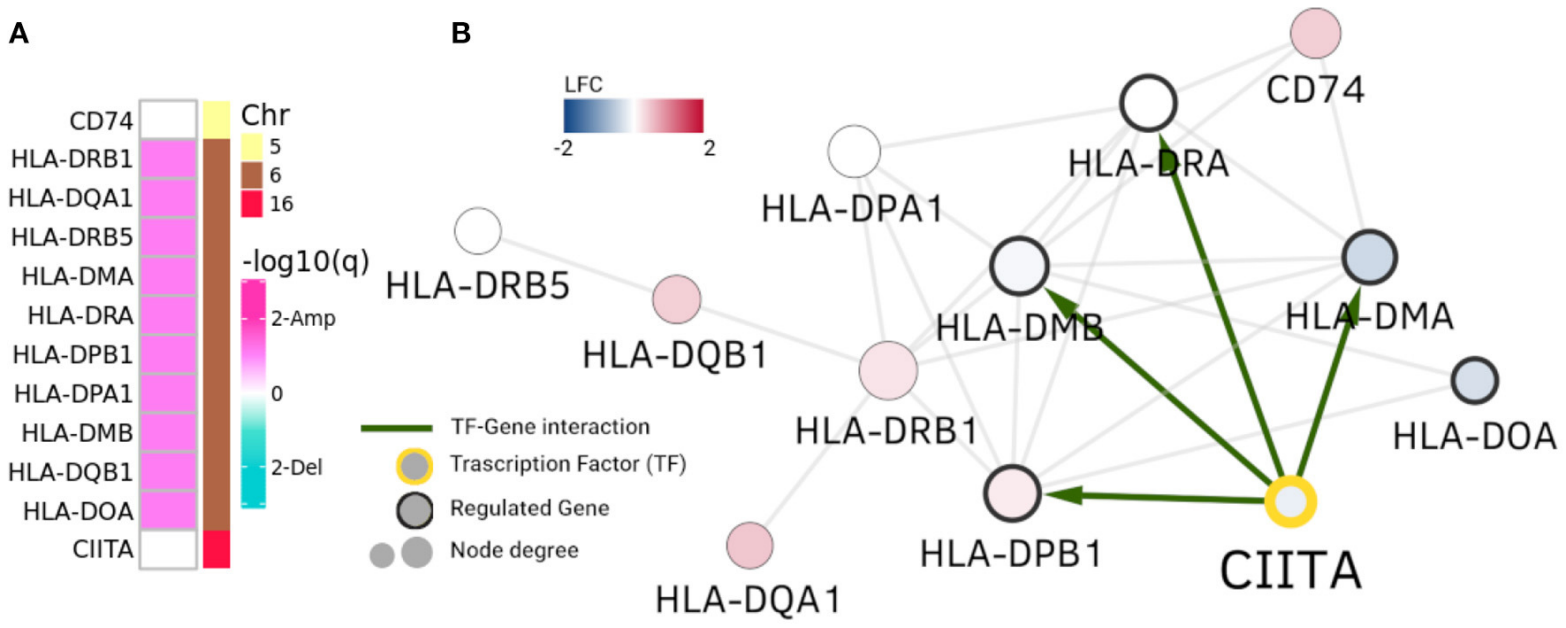
HLA-DRB1 community. In this picture, analogue to Figure 3, HLA-DRB1 community is depicted. Panel **(A)** displays amplification/deletion peaks in genes in the community and panel **(B)** shows differential expression and regulatory interactions. Genes in Chr6 (MHC class II genes) present amplifications. However, their differential expression is neither uniform nor with the same sign.

However, in the case of HLA-DRB1 community in Figure 4, we observe the opposite phenomenon: genes are not differentially expressed, but the ones that are placed in Chr6 belong to a clearly amplified region. This cluster is composed of MHC class II HLA genes. Interestingly, CIITA gene is a TF that regulates some of these human leukocyte antigen genes. As it can be observed in Figure 4, four of these genes have a CIITA binding site in their promoter region.

In this case CNAs and the CIITA regulation appear to exert a concomitant action with the observed copy number alterations to generate the community of MHC class II genes, independently of their differential expression. It is worth mentioning that CIITA (Class II Major Histocompatibility Complex Transactivator) is located at Chromosome 16, but clearly regulates the transcriptional and functional characteristics of HLA genes. The same representation for the RPL35 community is shown in Supplementary Figure 2. It is worth to stress that the HLA-DRB1 community in Luminal A GCN is almost identical to a community of the healthy GCN (Jaccard index = 0.916).

### 2.5. *cis-* Communities Are Enriched With Deletion Peaks

**Figure 5:**
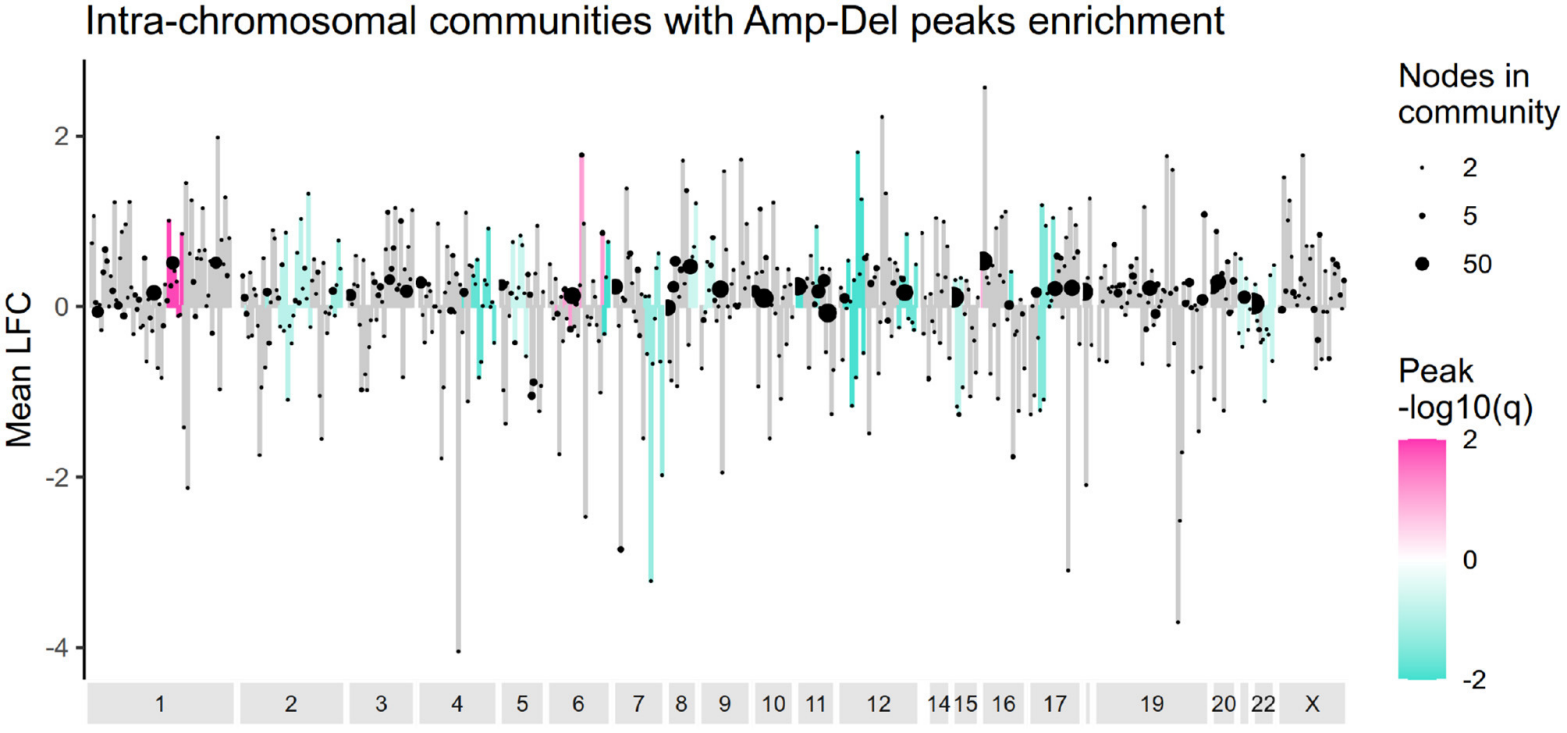
Amplification and deletion peaks overrepresentation in *cis-* communities. Intra-chromosomal communities are ordered according to the start of their first gene and the mean log2 fold change value for each community is plotted in the *y* axis. Communities enriched with amplification or deletion peaks are colored in pink or turquoise, accordingly. Ending dots indicate size of each community.

The presence of deletion and amplification peaks, and their effect in gene altered expression was also evaluated for *cis-* communities. Figure 5 presents the results of an overrepresentation analysis where GISTIC2 peaks were analyzed. As it can be observed, communities are mostly enriched with deletion peaks, and their effect in the average log2 fold change in *cis-* communities varies. Supplementary Figure 3 presents the entire set of alterations in these communities.

The pattern of amplification in the q arm of chromosome 1 and deletion in chromosome 16q, previously reported in a subset of Luminal A tumors (Ciriello et al., 2013) is also observed here. However, no other alteration matched that particular study. Luminal A tumors tend to have the lowest frequency of CNAs among breast cancer subtypes (Gatza et al., 2014), and as evaluated by our methodology, amplification and deletion peaks do not *a priori* determine the formation of *cis-* communities.

It is important to mention that copy number alterations are a key element affecting the gene expression of large sections of the genome (Freeman et al., 2006; Redon et al., 2006; McCarroll and Altshuler, 2007), specially in cancer (Shlien and Malkin, 2009; Lachmann, 2016; Shao et al., 2019). A large part of a chromosome being altered by a gain or loss of copy number, will trigger an equally abrupt change in several genes along that portion of the genome.

### 2.6. *cis-* Communities Are Not Bound by CTCF Binding Sites

The three-dimensional structure of DNA is another regulator of gene expression in eukaryotic cells. Regions with active transcription are characterized by open chromatin, whereas closed chromatin indicates regions of inactive or repressed transcription (Achinger-Kawecka et al., 2016; Corces and Corces, 2016). Furthermore, the regulatory effect of regions, such as enhancers and promoters, usually requires the formation of long distance chromatin loops that bring together distant genomic loci. These loops are maintained and regulated by architectural proteins, such as CTCF and cohesin, among others (Achinger-Kawecka and Clark, 2017; Pugacheva et al., 2020). Given the fact that CTCF proteins are able to modify the chromatin landscape, they may be underlying the appearance of a large amount of *cis-* communities in breast cancer.

To evaluate the role of CTCF in the appearance of *cis-* clusters of genes in the Luminal A breast cancer gene co-expression network, we calculated the number of CTCF binding sites at the boundaries of *cis-* communities. This was done using a previously reported dataset containing Chip-seq peaks in MCF7 cells, a Luminal A breast cancer cell line (Fiorito et al., 2016).

The number of binding sites in a window of 50k base pairs before the first gene and after the last one in a community was compared to the average number of binding sites in same size windows spanning the community region (see Methods). The distribution of these binding sites is shown in Supplementary Figure 4. No significant difference was found in the distribution of the number of binding sites in the boundaries and the middle sections of the communities. Actually, out of the 416 *cis-* communities with at least one CTCF binding site associated, only 197 had more binding sites at the boundaries than in middle regions.

### 2.7. Loss of Long-Distance Co-expression Does Not Depend on the Correlation Measure

**Figure 6:**
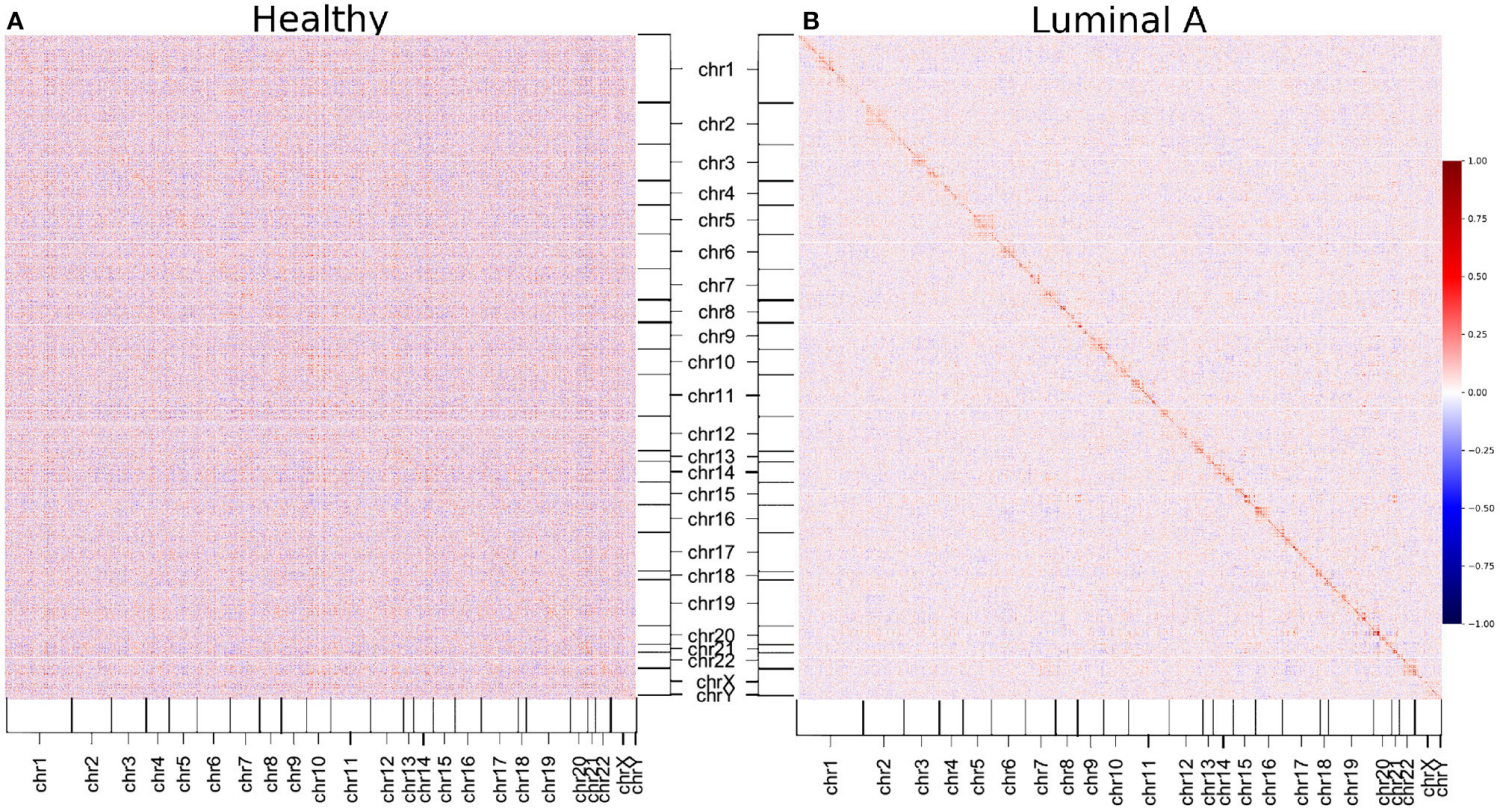
Pearson correlation matrices of healthy and Luminal A phenotypes. Correlation between all gene couples of each phenotype are depicted. The color code corresponds to the correlation value. **(A)** Healthy matrix. **(B)** Luminal A matrix. As in the case of mutual information-derived networks, higher correlation values in Luminal A occur between genes from the same chromosome (close to the diagonal).

We decided to construct GCNs for Luminal A and healthy phenotypes using Pearson correlation, to observe whether the phenomenon of loss of long-distance co-expression was maintained using other correlation measure. The results can be observed in the form of a heatmap in Figure 6. There, genes are placed according to its position in the chromosome. The color of the heatmap is proportional to the correlation value. The results show that, as observed with mutual information-inferred networks, the highest correlation values occur between genes from the same chromosome.

Additionally, it can also be appreciated that the Pearson correlation values are in general higher in the healthy matrix than in the Luminal A breast cancer one (except for those values close to the diagonal, which represent *cis-* interactions).

### 2.8. Loss of Long-Distance Co-expression Does Not Depend on the MI Threshold Value

Setting a threshold on the weight of edges so as to discard edges with strength less than a certain value is a well-known open problem in graph theory and network science. Determination of this threshold can be made by choosing among a number of methods. For instance, if an accurate measure of the signal-to-noise ratio in the correlations of the data under consideration can be obtained, one possible way to set the threshold is by allowing all edges valued above the noise-level. In most practical applications, however, this is not feasible.

**Figure 7:**
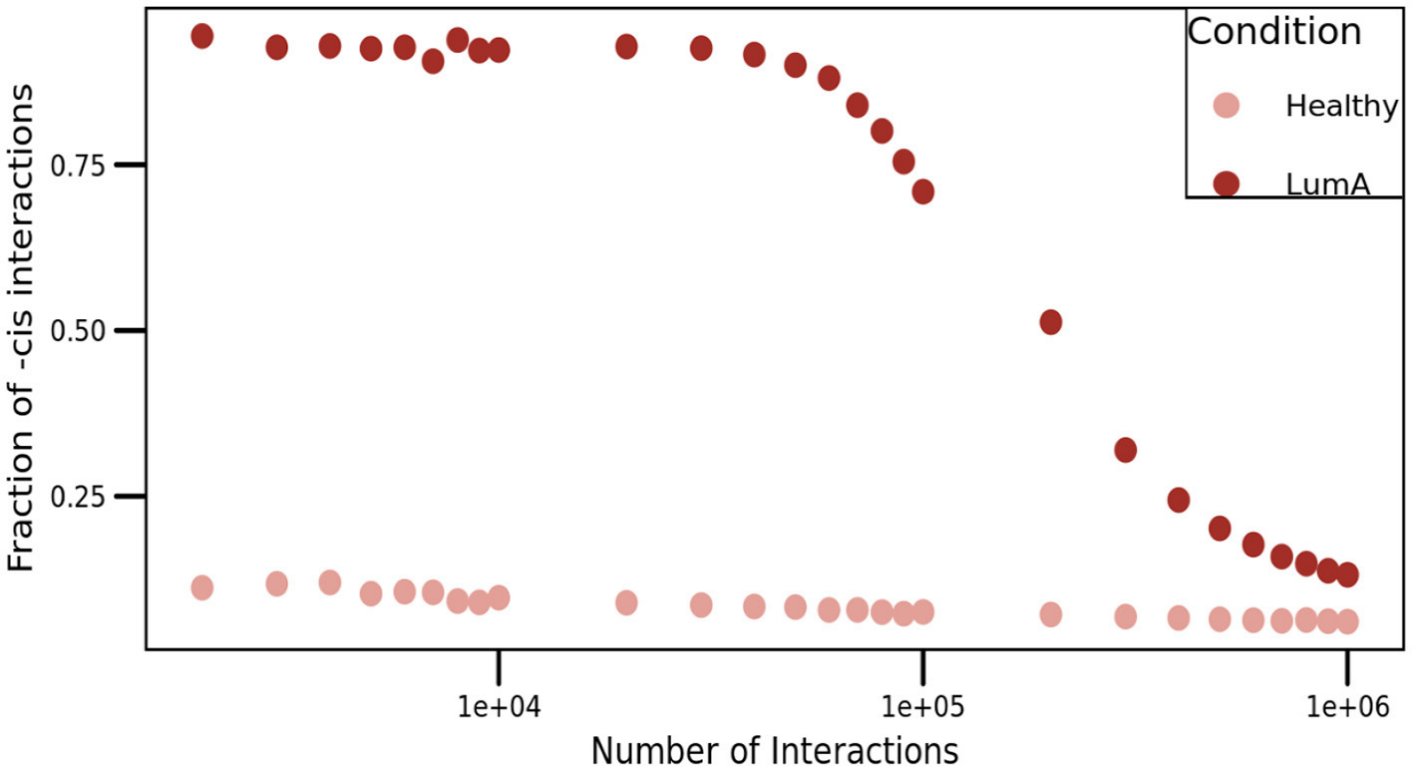
Proportion of *cis-* interactions at different network sizes. This figure shows the fraction of intra-chromosome interactions (Y-axis) for healthy (pale pink), and Luminal A (brown) GCNs. X-axis represents the number of edges in each network, ranging from the top-1,000 to the top-1,000,000 links, i.e., three orders of magnitude.

To overcome this situation, we presented a comparison of *cis/trans* proportion in both networks. For this purpose, we constructed networks with different threshold values, ranging from the top-1,000 to the top-1,000,000 higher edges (Figure 7). As it can be appreciated in the figure, the proportion of *cis-* interactions is always higher in Luminal A network than in the healthy GCN.

**Figure 8:**
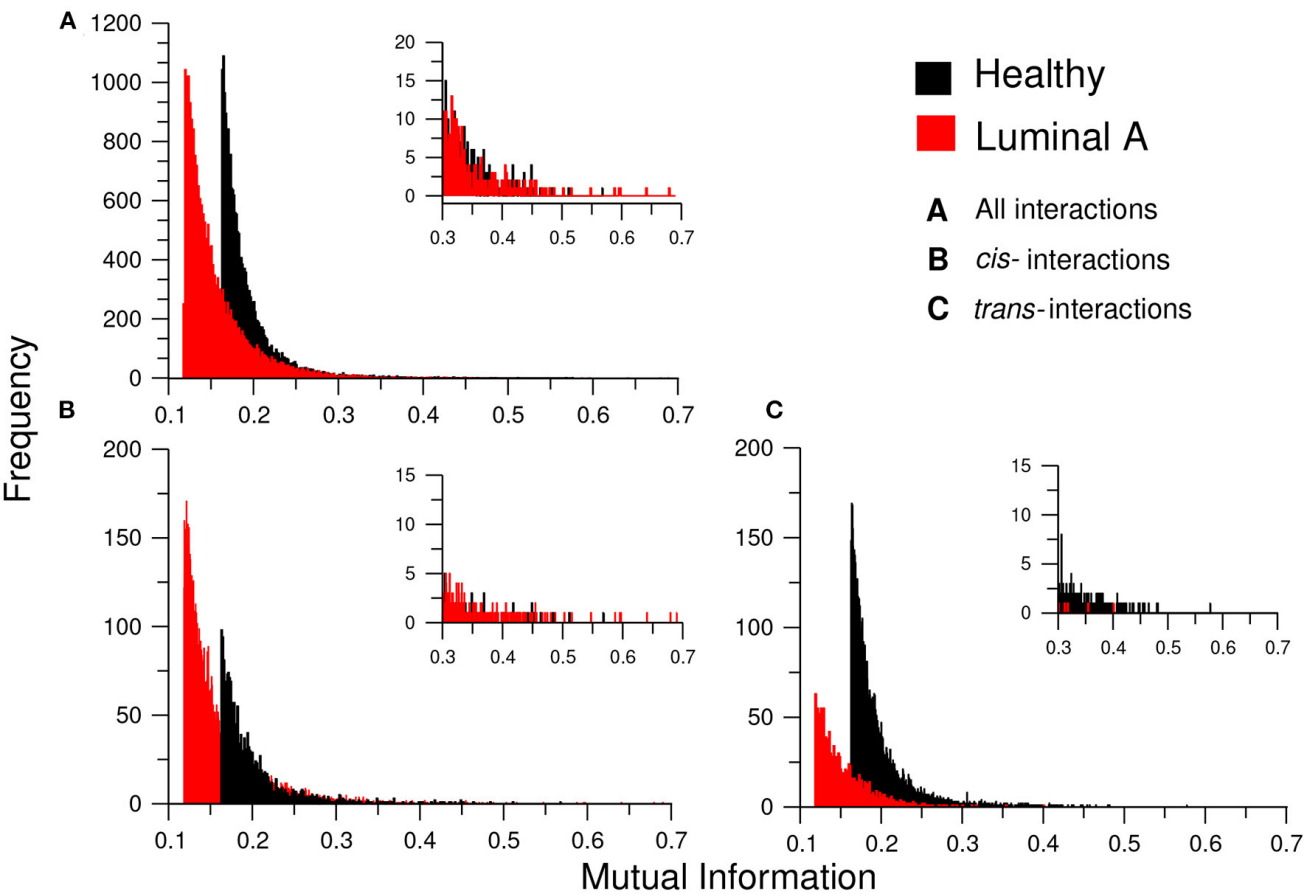
Distribution of MI values in the GCNs. This plot shows the histograms for the MI values of the healthy (black) and Luminal A (red) GCNs. **(A)** The total of MI values. **(B)** Only *cis-* edges, **(C)** Only *trans-* interactions. Each histogram also contains an inset with a zoom of the highest interactions for each condition. Notice the absence of *trans-* interactions in the Luminal A case in the inserts of **(B,C)**; this reflects the loss of *trans-* co-expression in the cancer GCN.

Additionally, to assess the influence of the MI threshold value in the phenomenon of loss of long-distance co-expression in Luminal A breast cancer, we observed the distribution of MI values in both networks. We constructed (a) the histograms of all interactions (20,217) in both networks, (b) the histograms for only *cis-* interactions, and (c) the histogram for *trans-* edges in both phenotypes (Figure 8). There, it can be observed that independently of the threshold, healthy interactions have higher MI values.

The above mentioned result coincides with the one presented in the matrices of Figure 6. Correlation values (independent on the correlation measure), are in general higher in the healthy phenotype than in cancer, but for a subset intra-chromosome interactions.

Complementarily, in Figure 8 we inserted a zoom of those histograms in the higher MI value region (0.3–0.7). There, it is shown that for *cis-* interactions, the Luminal A network has more and higher interactions in the highest values; conversely, for the *trans-* interactions, the higher and more abundant links are observed in the healthy phenotype.

We have shown previously that the threshold value is not determinant to observe the loss of long-distance co-expression in other clear cell renal carcinoma (Zamora-Fuentes et al., 2020), as well as in lung cancer (Andonegui-Elguera et al., 2021). We have demonstrated for these cancer GCNs that the particular value of the threshold, affects the size and sparsity of the networks as expected. However, the proportion of inter- and intra-chromosomal links remains largely unchanged.

### 2.9. Implications of Network Topology in the Context of Luminal a Breast Cancer

We have shown that in Luminal A breast cancer, the already mentioned *loss of trans- co-expression* is not as strong as in other breast cancer subtype GCNs, but the effect is perceived. Actually, several *trans-* interactions appear in the top co-expressed pairs. Luminal A GNC topology allows us to:

- identify functional communities (mostly *trans-*)
- differentiate enriched functions between healthy and cancer GCNs
- observe mechanisms that may influence the appearance of this loss of long distance co-expression
- observe specific differential expression patterns depending on the community

The identification of significant biological processes, associated with particular sets of highly co-expressed genes is one of the most relevant improvements of using network topology to analyze the functional implications of RNA-Seq-based genome-wide multi-sample sets for a given phenotype. The use of network communities improves the specificity of the enrichment analysis over using the whole genome or using differentially expressed genes.

The number of enriched processes in *cis-* communities is significantly lower than the ones associated with *trans-* communities, given the total number of communities for each type. However, the functions that are significant for *cis-* communities, are also relevant for cell maintenance. For instance, HOXA community, whose genes are relevant for organism development. These genes are found together in chromosome 7p15.2, and they are all underexpressed. Analogously, the protocadherin cluster is found to be related to cell adhesion, which is one of the non-shared processes between Luminal A GCN and the healthy GCN (Supplementary Material 1).

From the alluvial diagram of Figure 2 it can be observed that out of the 11 enriched *cis-* communities, 6 correspond to HOX and protocadherin clusters. This could be an indicative of the importance of the conjugated action that these set of genes may have for the phenotype. Additionally, these clusters appear with the same differential expression trend.

## 3. Concluding Remarks

Based on the previous analysis, we may conclude that for the establishment of the regulatory program observed in the Luminal A subtype gene co-expression network, compared with the healthy GCN, several DNA modifications and regulatory elements must participate. DNA modifications (copy number alterations, transcription factor regulation, CTCF binding sites) should exert, to at certain extent, influence over the gene co-expression interactions. Additionally, differential gene expression is a relevant element to take into account, specially for *trans-* communities. We can establish that, for the manifestation of the *loss of trans- co-expression* in cancer it is not only necessary to observe separately differential gene expression, transcription factor regulation, CNAs, or CTCF binding sites, but to take them all into account.

Other regulatory elements should also participate in modifying the co-expression patterns between a healthy and a cancer co-expression network: micro-RNA regulation (Drago-Garćıa et al., 2017; de Anda-Jáuregui et al., 2018), topologically associated domains and their boundaries (Rafique et al., 2015; Achinger-Kawecka et al., 2020; Khoury et al., 2020), long non-coding RNAs (Hung et al., 2011; Zhang et al., 2019), the methylation profiles (Paz et al., 2003; Hernández-Lemus et al., 2019), among others, might delineate these imbalance between *cis-* and *trans-* genetic relationships.

More investigation regarding the aforementioned elements is also important in order to have an integral picture of the regulatory landscape in the cancer genome, and provide hypotheses that could explain the phenomenon of loss of long distance genetic interactions in cancer.

It is likely plausible that the *loss of trans- co-expression* observed in breast cancer (and breast cancer molecular subtypes) responds to a physical/mechanical principle in which the transcriptional machinery is somehow altered. Recently, we have observed the loss of long distance co-expression in clear cell renal carcinoma (Zamora-Fuentes et al., 2020), and in lung adenocarcinoma, as well as in squamous cell lung cancer (Andonegui-Elguera et al., 2021).

The ubiquity of this disruption of the *normal* transcriptional landscape led us to hypothesize that the physical principle behind this global alteration is the same in all of these cancer tissues. The consistency and relevance of this loss could be considered as a possible emergent hallmark of cancer. Further investigation toward this particular issue must be achieved beforehands, however, further investigation is required.

## 4. Methods

### 4.1. Databases

Gene expression values for Luminal A and Healthy samples were retrieved from our previous publication (García-Cortés et al., 2020), with RNA-seq data obtained from The Cancer Genome Atlas (TCGA) breast invasive carcinoma dataset (Tomczak et al., 2015), downloaded from the Genomic Data Commons (GDC) Data Portal. The GDC Data portal case identifiers for Luminal A were use to download “Masked Copy Number Segment Files” for the GISTIC2 pipeline. The Chip-seq data was downloaded from the Gene Expression Omnibus dataset GSE85106 (Fiorito et al., 2016), and only the control sample for CTCF was used. The Homo sapiens genes promoter dataset from the Gene Transcription Regulation Database (GTRD) (Yevshin et al., 2018) was used to identify transcription factors and their regulatory interactions.

### 4.2. Data Processing

As detailed in García-Cortés et al. (2020), 113 samples for Healthy tissue and 1,102 cancer samples were acquired and pre-processed to *log*_2_ normalized gene expression values. After applying the PAM50 algorithm using the Permutation-Based Confidence for Molecular Classification (Fresno et al., 2017) as implemented in the pbcmc R package (Fresno et al., 2016), and multidimensional noise reduction using ARSyN R implementation (Nueda et al., 2012), 217 samples for Luminal A breast cancer were identified.

The “Masked Copy Number Segment Files” were downloaded from GDC and integrated into one segmentation file to run gistic2 (Mermel et al., 2011). The parameters suggested in the Copy Number Variation Analysis Pipeline from GDC and the GDC reference sequence, and markers file were used. The identified amplification and deletion regions in the lesions output file with 0.99 confidence were re-mapped to keep genes spanned entirely by peaks.

### 4.3. Network Construction

The ARACNE (Margolin et al., 2006) algorithm was used to calculate mutual information (MI) to quantify statistical dependence between pairs of genes. The method associates a significance value (*p*-value) to each *MI* value based on permutation analysis, as a function of the sample size. Only the highest interactions in terms of their statistical significance (*P* ≤ 1*e*^−8^) were kept for further analysis. The total number of interactions in the Luminal A and the Healthy network were reduced to 20,127, the number of significant interactions in the Healthy network.

### 4.4. Community Detection and Assortativity Calculation

Four community detection algorithms were evaluated: Fast Greedy (Clauset et al., 2004), Infomap (Rosvall and Bergstrom, 2008), Leading Eigenvector (Newman, 2006), and Louvain (Blondel et al., 2008; Rahiminejad et al., 2019). MI values were used as link weights. Their implementation in the igraph (Csardi and Nepusz, 2006) R package was used. Algorithm results were compared using the Jaccard index, a coefficient that measures similarity between two finite sets, defined as the size of their intersection divided by the size of their union. Genes in a community constitute a set and all communities identified by one algorithm were compared against communities identified by another one. The same approach was used to compare the set of GO terms associated per community in the overrepresentation analysis.

(1) $$\begin{array}{l} J\left(C_{1},C_{2}\right)=\frac{\left(C_{1}\cap C_{2}\right)}{\left(C_{1}\cup C_{2}\right)} \end{array}$$

To calculate chromosomal assortativity, the chromosome location for each gene was used. For each community, the number of links joining genes in the same chromosome (*-cis* links) minus the number of links joining genes in different chromosomes (*-trans* links), was divided by the total number of links in the community. Expression assortativity was calculated in the same manner, using the log2 fold change sign to classify genes into overexpressed or underexpressed as the assortativity attribute.

$$\begin{array}{l} \begin{array}{c} ASS_{chr}=\\ \frac{\left|\left\{\left\{x,y\right\}|x,y\in C_{i}\text{ and }x\text{.chr}=y\text{.chr}\right\}|-|\left\{\left\{x,y\right\}|x,y\in C_{i}\text{ and }x\text{.chr}\neq y\text{.chr}\right\}\right|}{\left|\left\{\left\{x,y\right\}|x,y\in C_{i}\right\}\right|}\\ C_{i}=\text{community }i\text{ in network}. \end{array} \end{array}$$

### 4.5. Overrepresentation Analysis

The enrichGO function from the clusterProfiler (Yu et al., 2012) R package was used to identify over-represented or enriched terms in the Biological Process category in Gene Ontology (GO). Enrichment was performed for communities with five or more genes and GO terms with a minimum size of ten were retained. Genes in the original expression matrix defined the universe set. Terms with adjusted *p*-value below 0.005 using the Benjamini and Hochberg method for multiple testing were kept. The overrepresentation analysis for amplification and deletion peaks was conducted using the generic function enricher from the same package. The same universe set was used and no size threshold for communities or peaks was defined. An adjusted *p*-value of 0.05 was set as cutoff.

### 4.6. Differential Expression Analysis

Differential expression analysis was performed as described in (Espinal-Enríquez et al., 2017). The limma package (Ritchie et al., 2015) in R was used to determine overexpressed or underexpressed genes, by adjusting a gene based linear model. An absolute difference of log2 fold change ≥0.5 and a *p*-value < 0.05 was set as threshold.

### 4.7. Transcription Factors Identification

The entire set of gene promoters in the smallest region available, [−100, +10] base pairs from starting site was downloaded from the Gene Transcription Regulation Database (GTRD) (Yevshin et al., 2018). For the selected communities, gene members that matched transcription factors (TF) in GTRD were extracted and their neighboring genes were compared to the set of annotated genes that had at least one binding site from that TF in the ChIP-seq data.

### 4.8. CTCFs

We took the CTCFs in genes and promoters in the *cis-* Luminal A network communities that were not in other genes or promoters. For the Inter-regional CTCFs, we took the ones that were in a region <50k bps from the extreme of the promoter and the extreme of the gene.

Once filtered, the binding sites were classified according to their location. CTCFs in gene bodies, promoters (+1,000, −500 bps) and intergenic region were identifies. Table 3 displays the classified binding sites for the complete dataset, as well as the binding sites present in genes comprising the Luminal A *trans-* communities. For the intergenic region, only CTCF binding sites in a window of 50k base pairs upstream the first gene and downstream the last one in *cis-* communities were kept.

**Table 3:** CTCF binding sites location classification.

	**Promoter**	**Gene body**	**Intergenic region**
Dataset	868	8,047	11,438
In Luminal A network	177	1,343	887

## Data Availability Statement

Publicly available datasets were analyzed in this study. This data can be found at: Genomic Data Commons Data Portal https://portal.gdc.cancer.gov/, Gene Expression Omnibus dataset GSE85106 Gene Transcription Regulation Database, http://gtrd.biouml.org/, Relevant data used, and the scripts to generate the results and figures can be found in the following repository: https://github.com/ddiannae/luma.

## Author Contributions

DG-C performed the computational analyses, developed and implemented the programming code, performed the pre-processing and low-level data analysis, made the figures, drafted the manuscript. EH-L contributed to the theoretical analysis, co-supervised the project, contributed to the writing of the manuscript. JE-E conceived and designed the project, co-supervised the project, discussed the results, drafted the manuscript. All authors read and approved the final version of the manuscript.

## Conflict of Interest

The authors declare that the research was conducted in the absence of any commercial or financial relationships that could be construed as a potential conflict of interest.

## Supplementary Material

Supplementary Material 1 Results of community detection algorithms evaluation.

Supplementary Table 1 List of Gene Ontology biological processes overrepresented in the Healthy and the Luminal A gene co-expression networks (GCN), as well as the shared enriched terms between both networks.

Supplementary Table 2 List of genes in the Luminal A GCN and the Healthy GCN with their chromosomal location, associated log2 fold change (LFC) value, and corresponding community for each algorithm.

Supplementary Figure 1 Differential expression in the Luminal A GCN. The NUSAP1 community is highlighted.

Supplementary Figure 2 RPL35 community. Left panel presents amplification and deletion peaks identified by GISTIC2, through pink and turquoise squares. Genes are ordered according to their corresponding chromosome. Right panel displays differential expression and regulatory interactions in genes in the community.

Supplementary Figure 3 Amplification and deletion peaks in *cis-* communities. Entire set of copy number alterations identified in intra-chromosomal communities. Genes are displayed according to their starting site.

Supplementary Figure 4 CTCF binding sites distribution over *cis-* communities. Biding sites at a distance of no more than 50,000 base pairs from a gene in the community are displayed.

## Abbreviations

CNA: copy number alteration
GCN: gene co-expression network
GTRD: gene transcription regulation database
LFC: Log2 fold change.
