# Supplementary material for: Luminal A Breast Cancer Co-expression Network: Structural and Functional Alterations
Source: Front Genet. 2021 Apr 20;12:629475. doi: 10.3389/fgene.2021.629475 (PMC8096206; doi:10.3389/fgene.2021.629475)
Supplement: Supplementary Material 1 — Results of community detection algorithms evaluation. [file Data_Sheet_1.pdf]

Community Detection Algorithms

Community detection was performed using the following algorithms implemented in the R package igraph. MI values were taken as link weights.

- Fast Greedy
- Infomap
- Leading Eigenvector
- Louvain

Community sizes comparison

The distribution of community sizes identified by each algorithm is displayed in Figure 1.

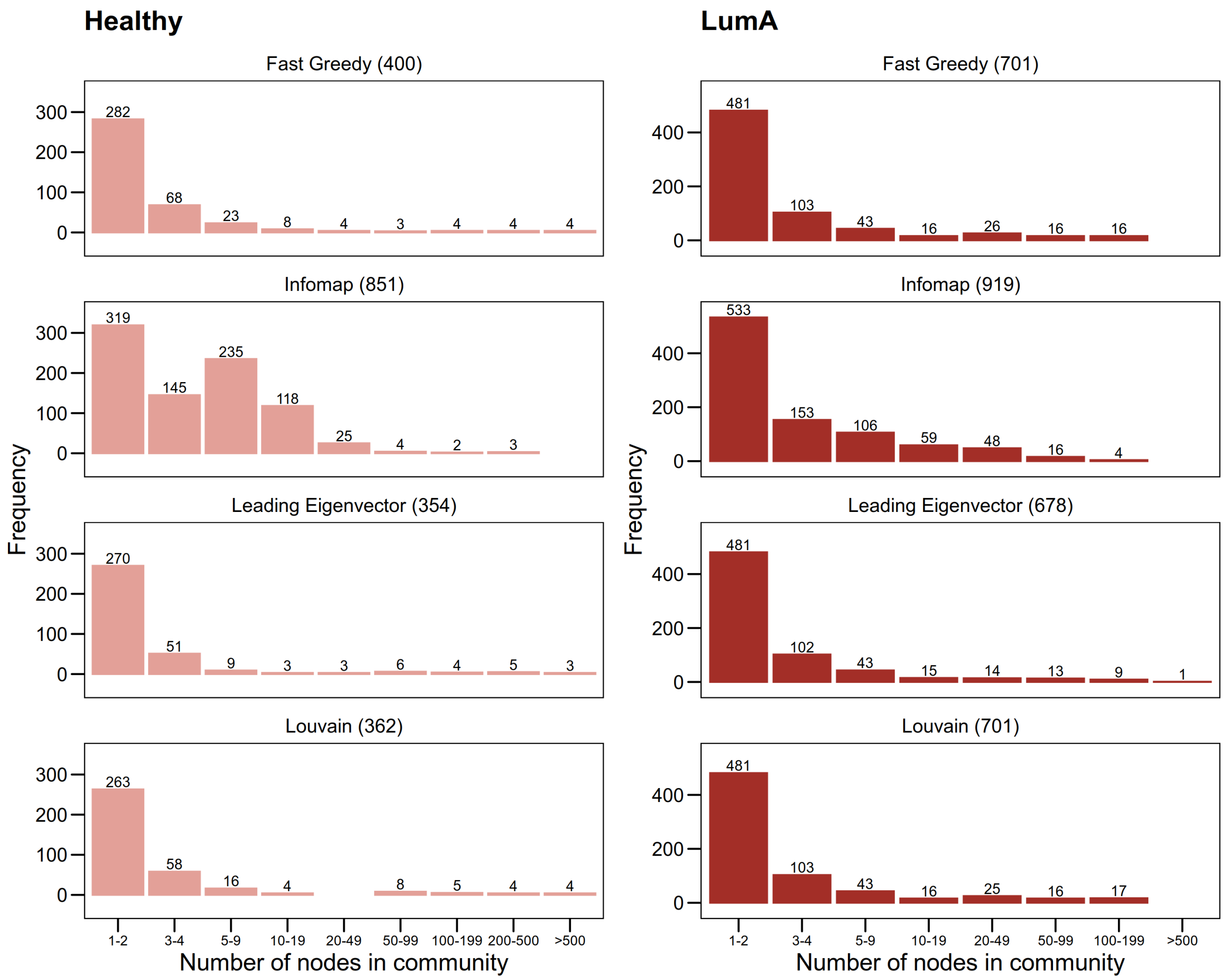

Figure 1: Distribution of community sizes identified by each algorithm.

Community sets comparison

To compare the similarity among communities, Jaccard indexes were computed for all communities from one algorithm against all communities from another one. As stated in the main text, Infomap algorithm presents the most dissimilar result, while the other algorithms present over 90% overlap in the identified communities.

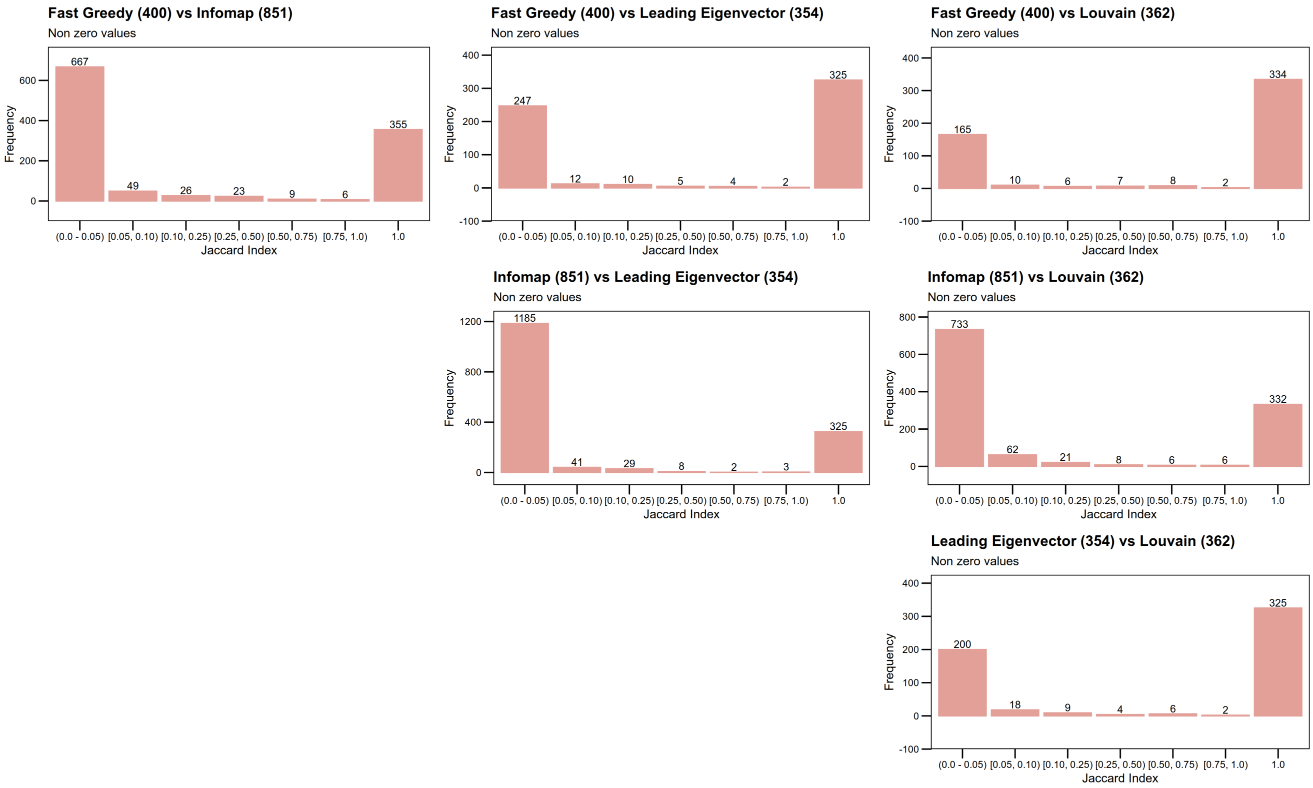

Figure 2: Jaccard indexes for Healthy communities

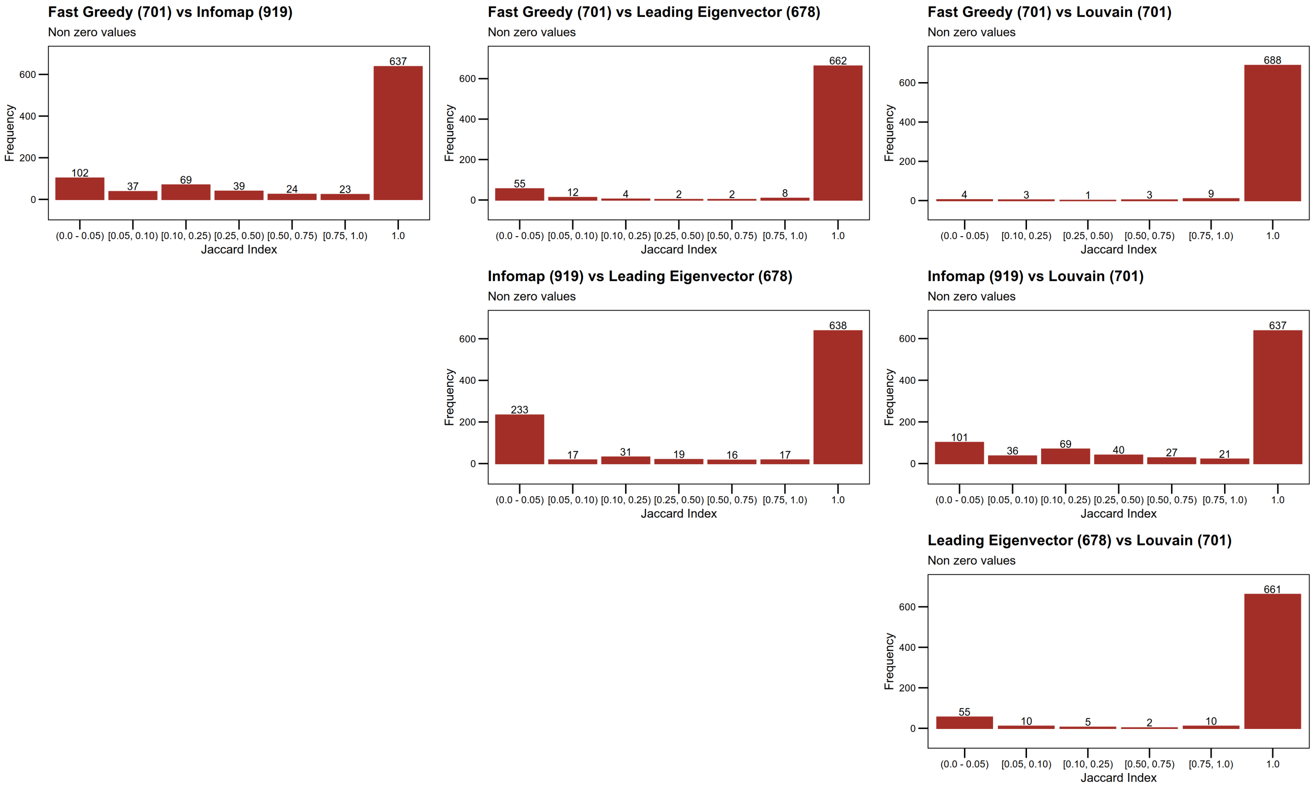

Figure 3: Jaccard indexes for Luminal A communities

Louvain algorithm communities

Table 1 in the main text displays Modularity values obtained for community detection in both networks. The Louvain algorithm presents the highest values.

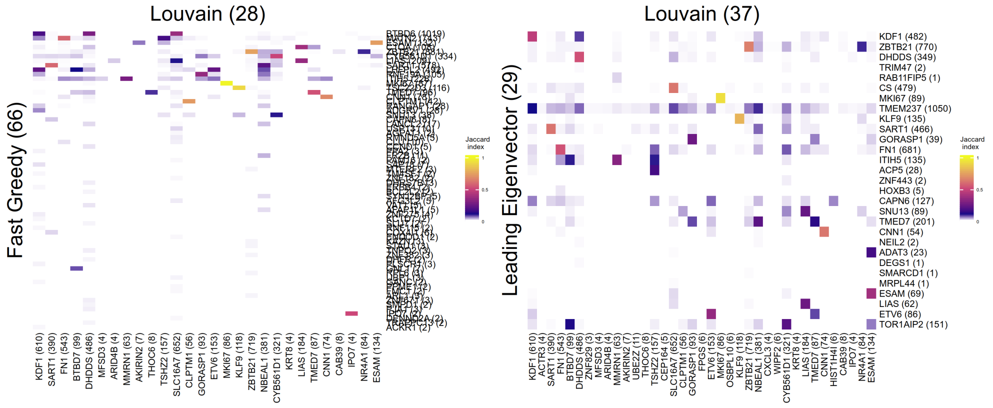

Figure 4: Jaccard index values less than 1 (communities with no complete overlap) for Healthy communities identified by Fast Gready, Leading Eigenvector and Louvain algorithms

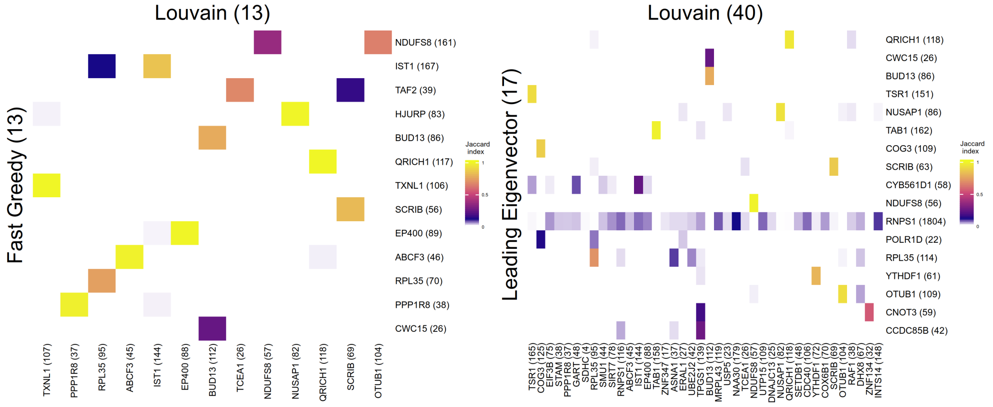

Figure 5: Jaccard index values less than 1 (communities with no complete overlap) for Luminal A communities identified using Fast Gready, Leading Eigenvector and Louvain algorithms

Chromosomal assortativity comparison

Chromosomal assortativity was calculated as the total number of intra-chromosomal links minus the number of inter-chromosomal links, divided by the total number of links in a community. The four algorithms used in the analysis present similar results.

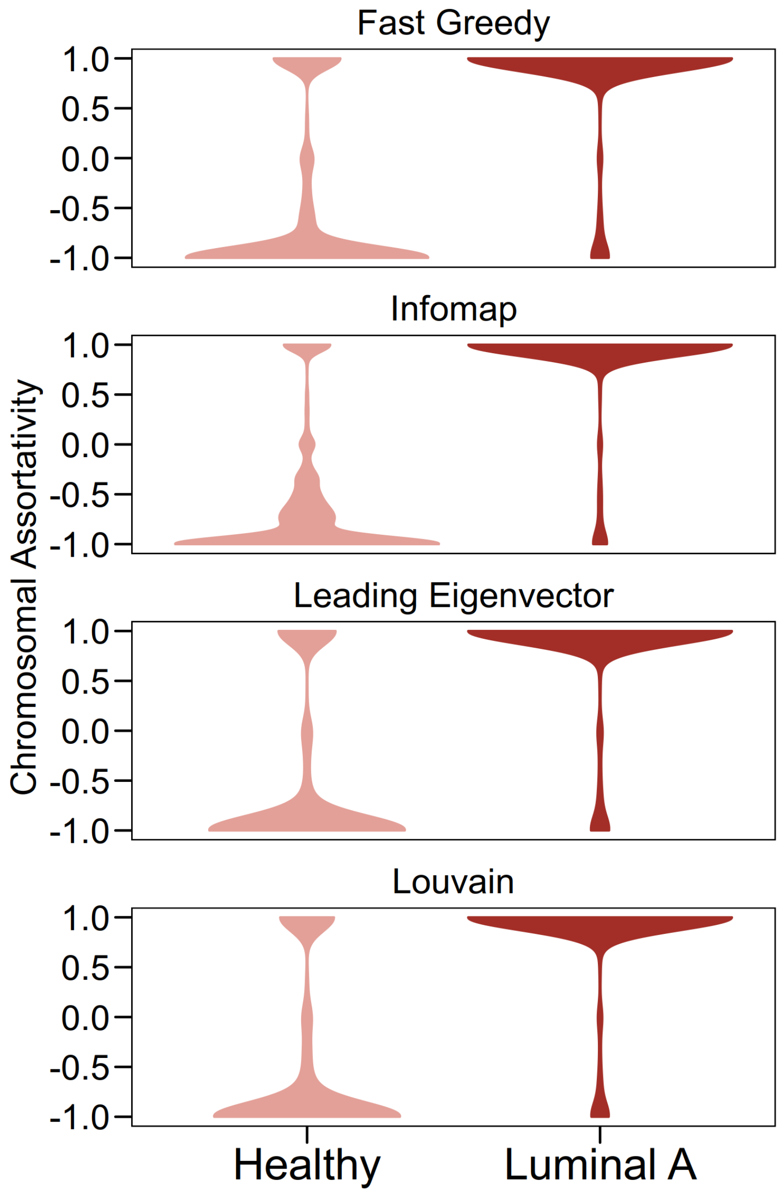

Figure 6: Chromosomal assortativity distribution in the Healthy and the Luminal A GCN, with communities identified by different algorithms.

Enrichment comparison

An overrepresentation analysis was performed per community using set of GO Biological processes. Names are given according to the gene with highest page rank value in the community. Again, Infomap presents the most dissimilar results, while other algorithms present almost a 1 to 1 mapping between pairs of enriched communities.

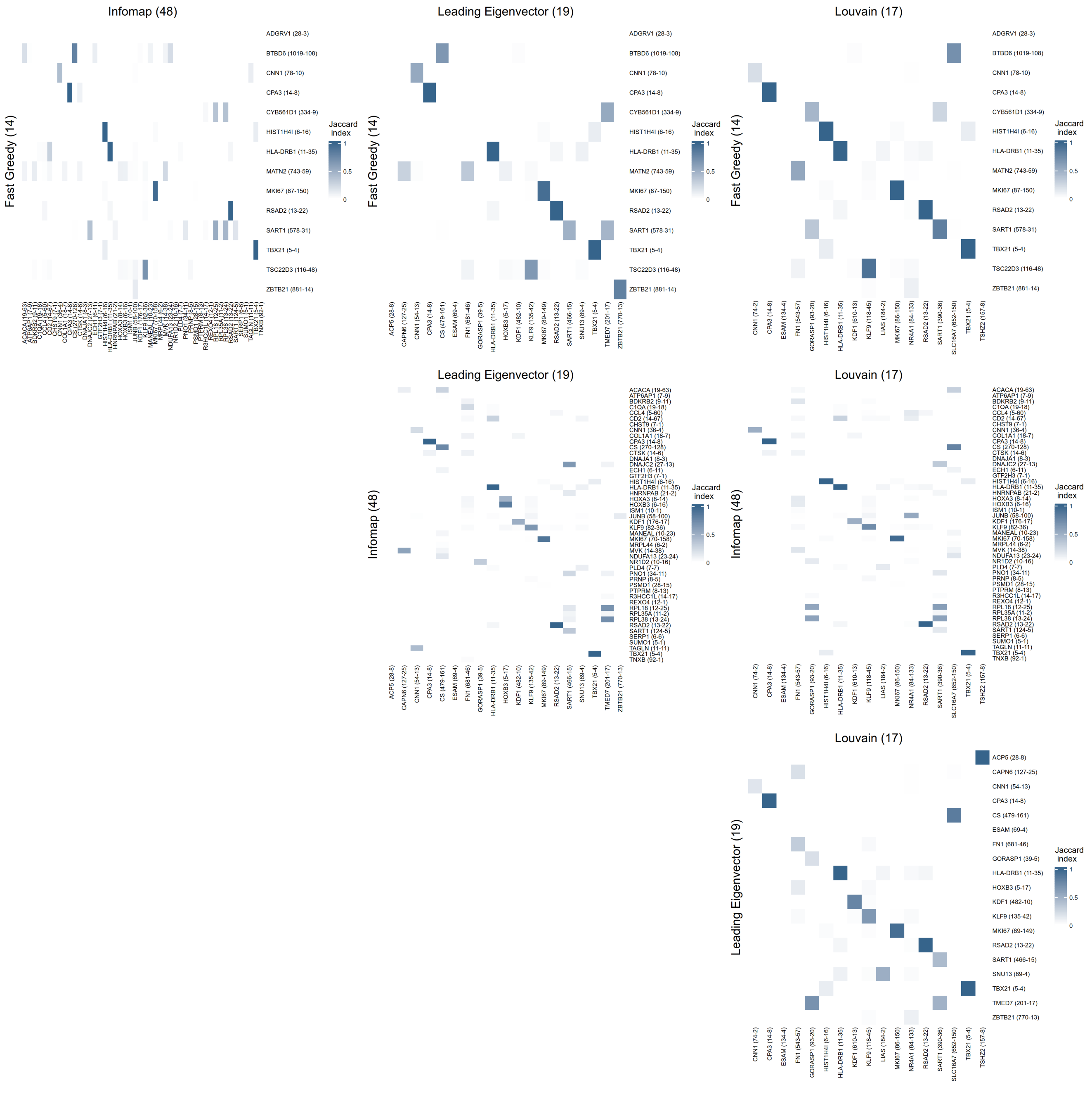

Figure 7: Jaccard indexes of the set of GO Biological Processes associated per community in the Healthy network, with communities detected by different algorithms. Inside parenthesis the total number of genes in the community and the number of associated GO terms are displayed.

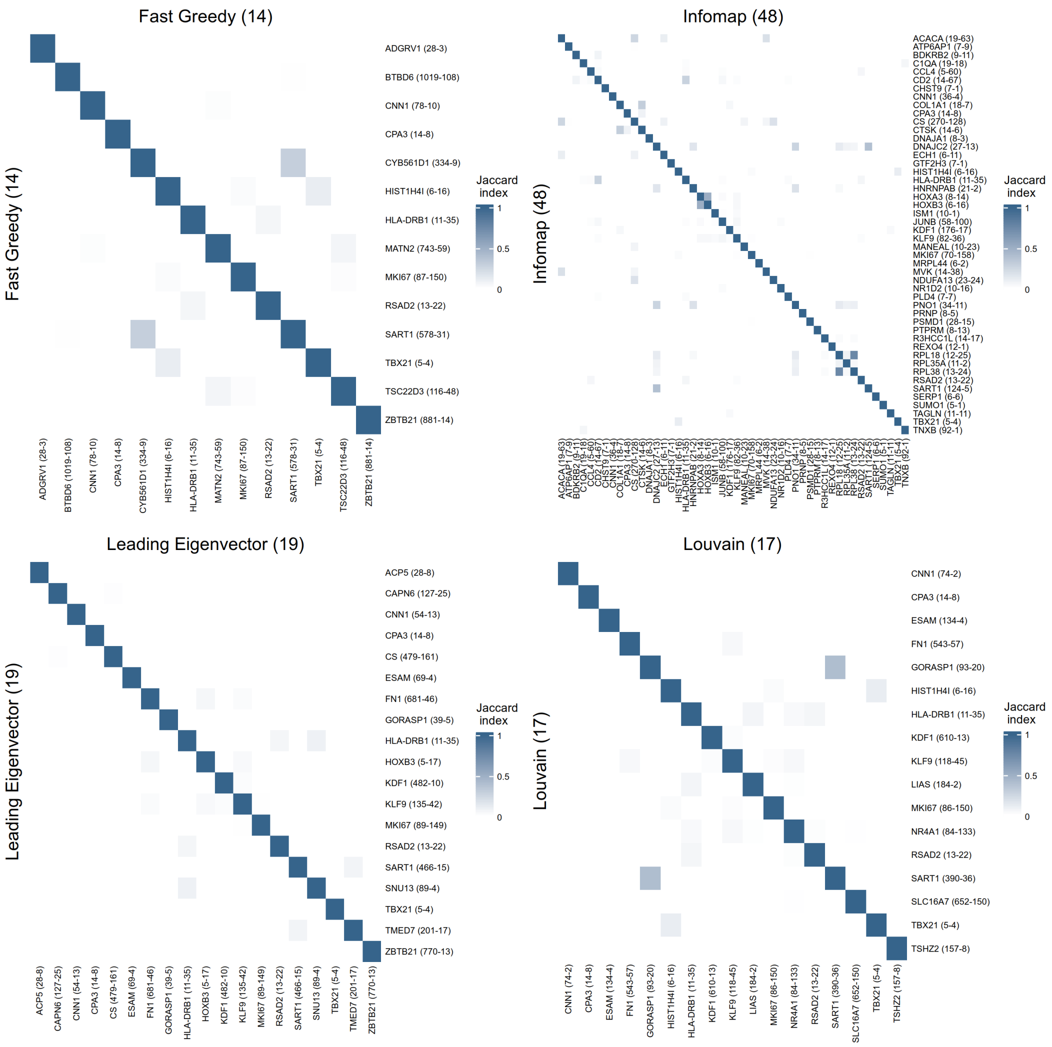

Figure 8: Jaccard indexes of the set of GO Biological processes associated per community in the Healthy network with communities detected by one algorithm. Inside parenthesis the total number of genes in the community and the number of associated GO terms are displayed.

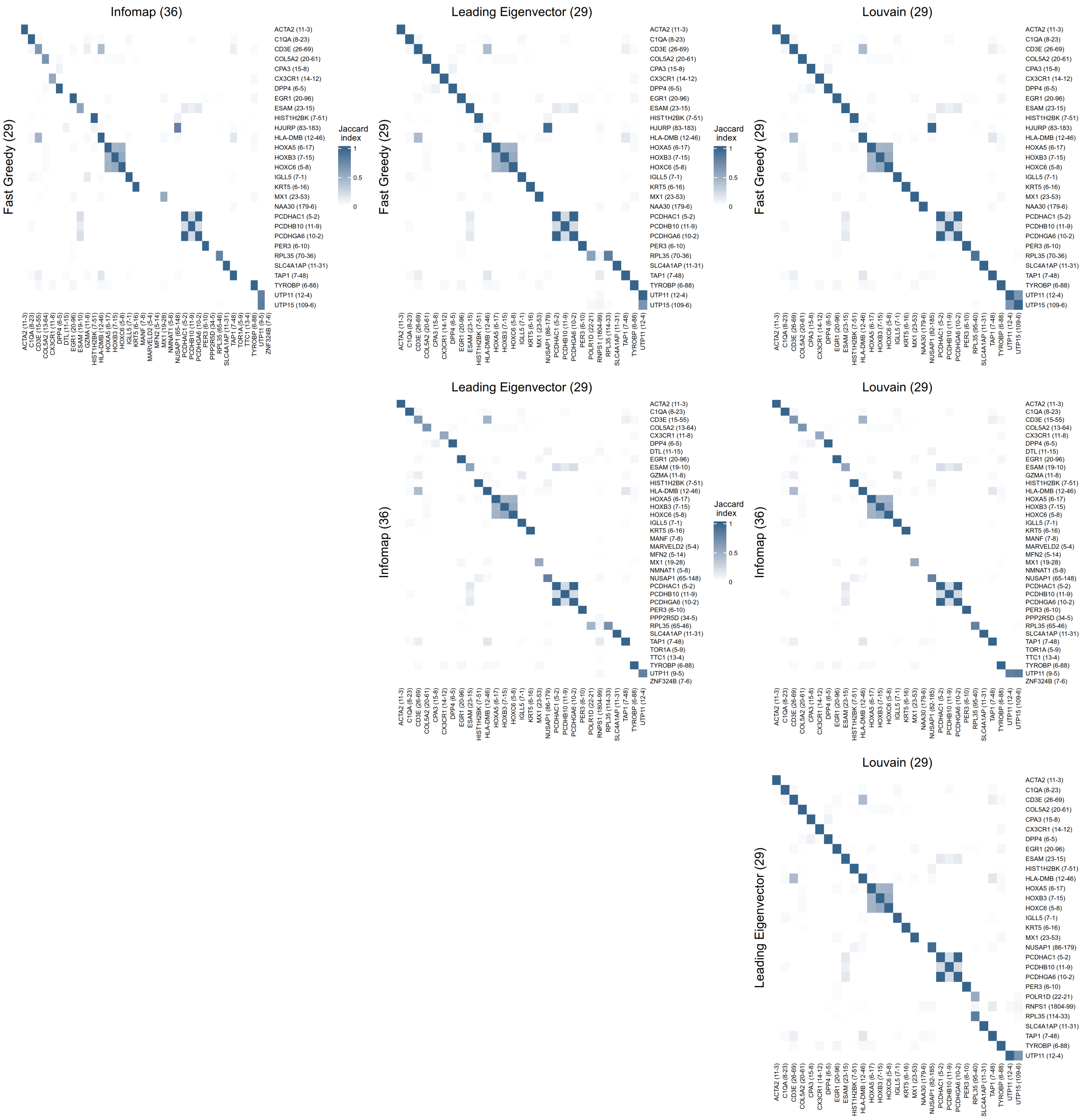

Figure 9: Jaccard indexes of the set of GO Biological processes associated per community in the Luminal A network with communities detected by different algorithm algorithms. Inside parenthesis the total number of genes in the community and the number of associated GO terms are displayed.

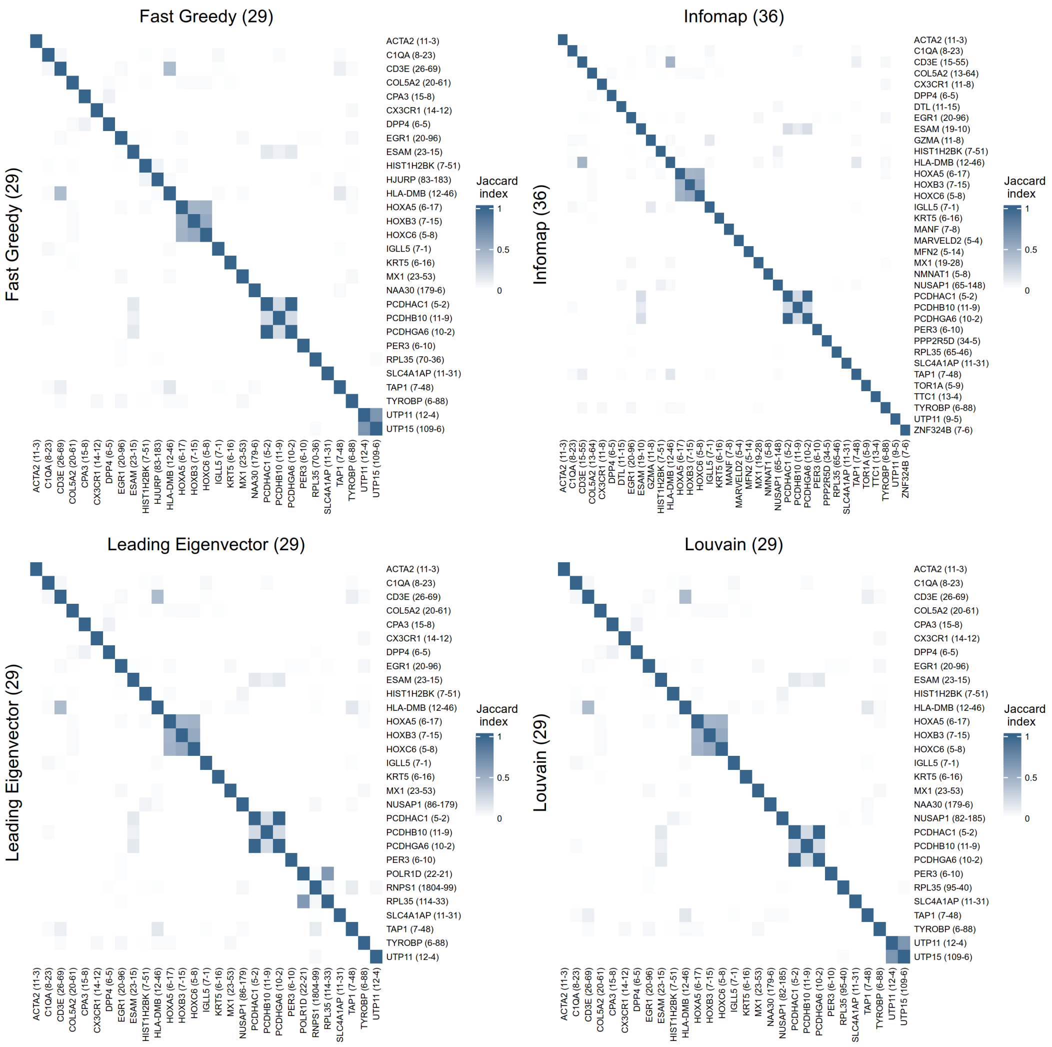

Figure 10: Jaccard indexes of the set of GO Biological processes associated per community in the Luminal A network with communities detected by one algorithm. Inside parenthesis, the total number of genes in the community and the number of associated GO terms are displayed.

Healthy vs Luminal A

Communities comparison

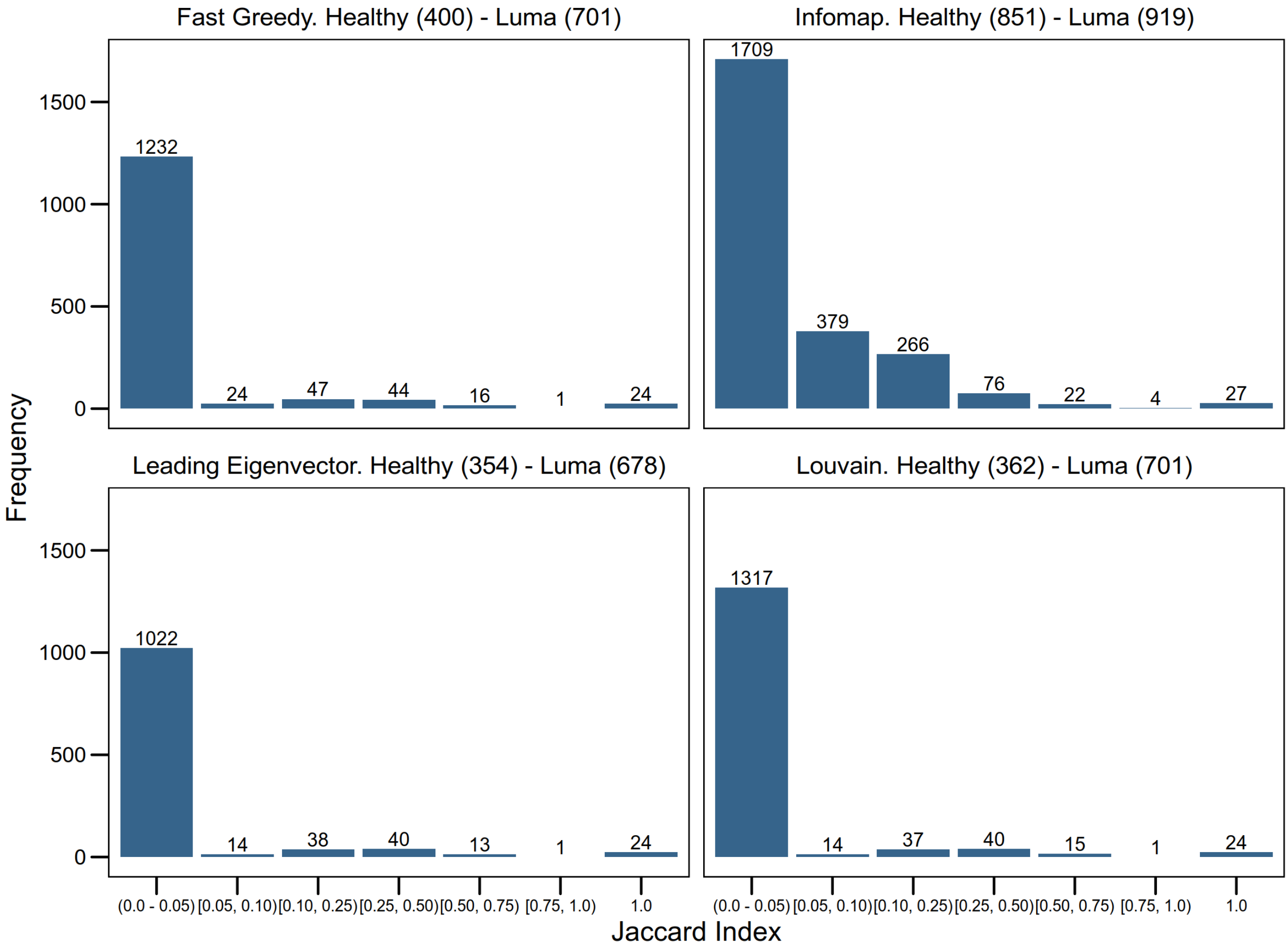

Figure 11: Jaccard indexes of the communities identified for each network by all algorithms. Communities with Jaccard index equal to 1 are mostly composed by two nodes, except for one community with three nodes. Titles contain the total number of communities identified by each algorithm.

Erichments comparison

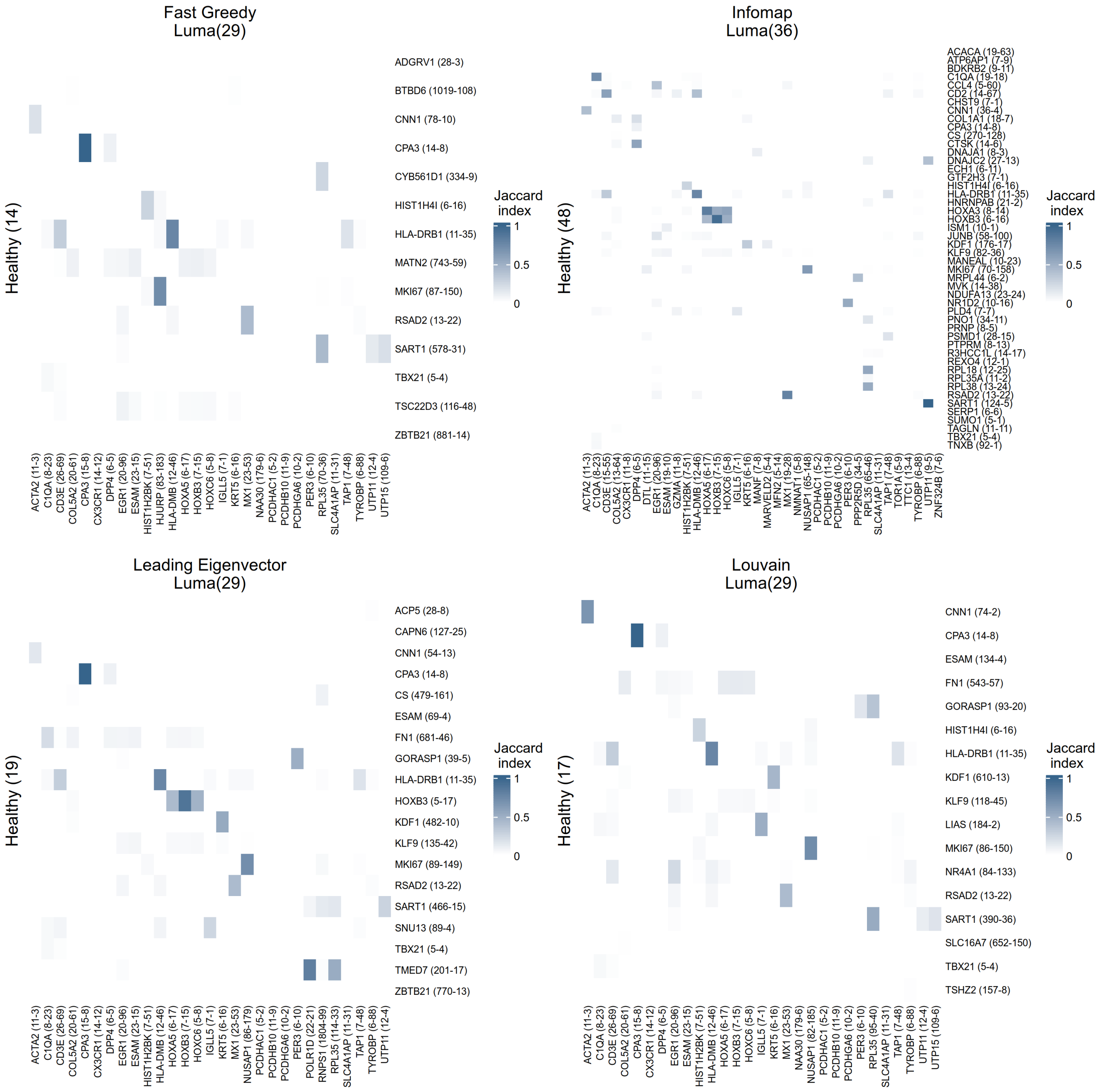

Figure 12: Jaccard indexes of the the set of GO Biological processes associated per community in the Luminal A network and the Healthy network identified by each algorithm. Inside parenthesis, the total number of genes in the community and the number of associated GO terms are displayed.
