# Supplementary figures and images for: Luminal A Breast Cancer Co-expression Network: Structural and Functional Alterations
Source: Front Genet. 2021 Apr 20;12:629475. doi: 10.3389/fgene.2021.629475 (PMC8096206; doi:10.3389/fgene.2021.629475)

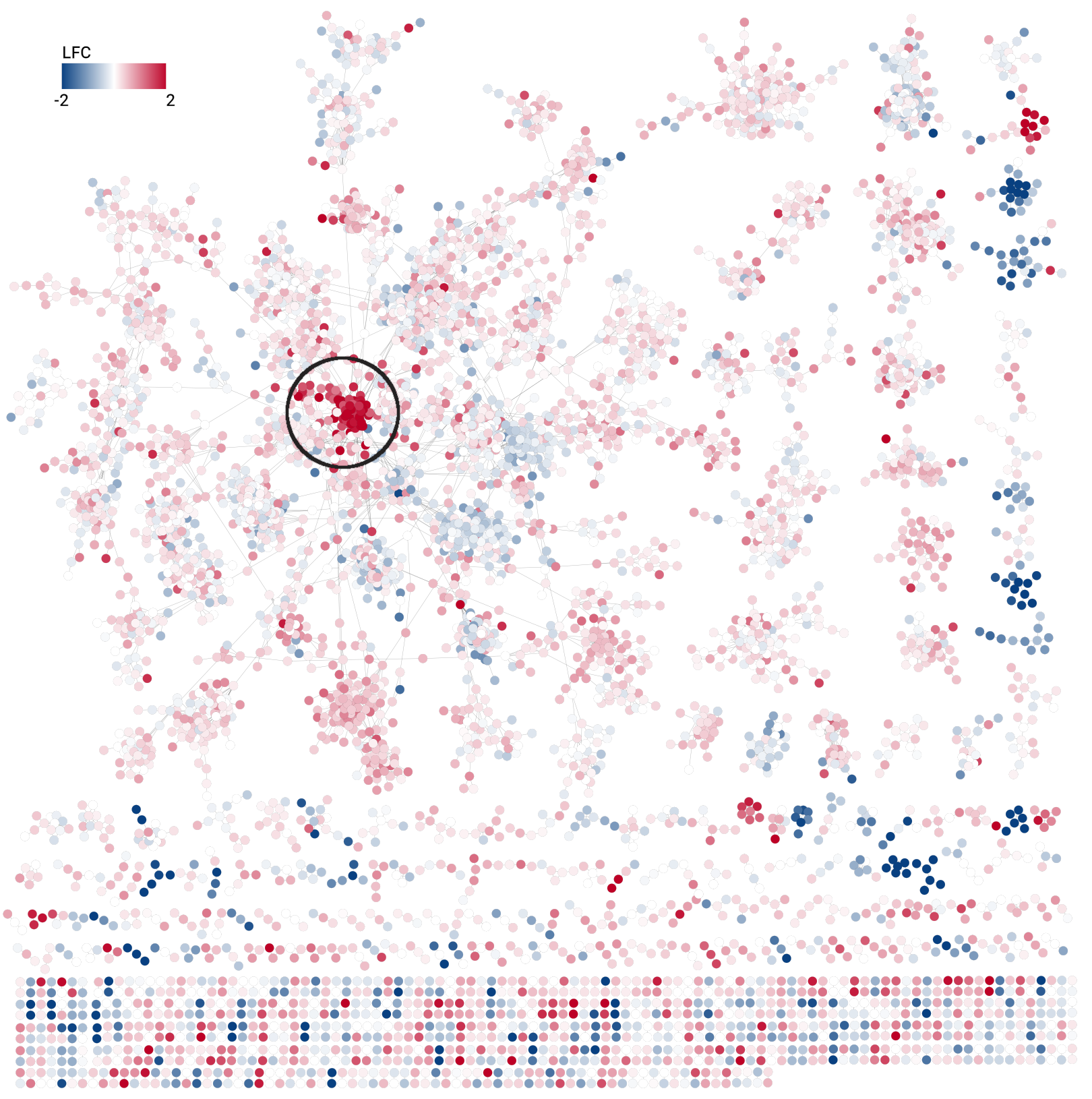

Supplement: Supplementary Figure 1 — Differential expression in the Luminal A GCN. The NUSAP1 community is highlighted. [file Image_1.PNG]

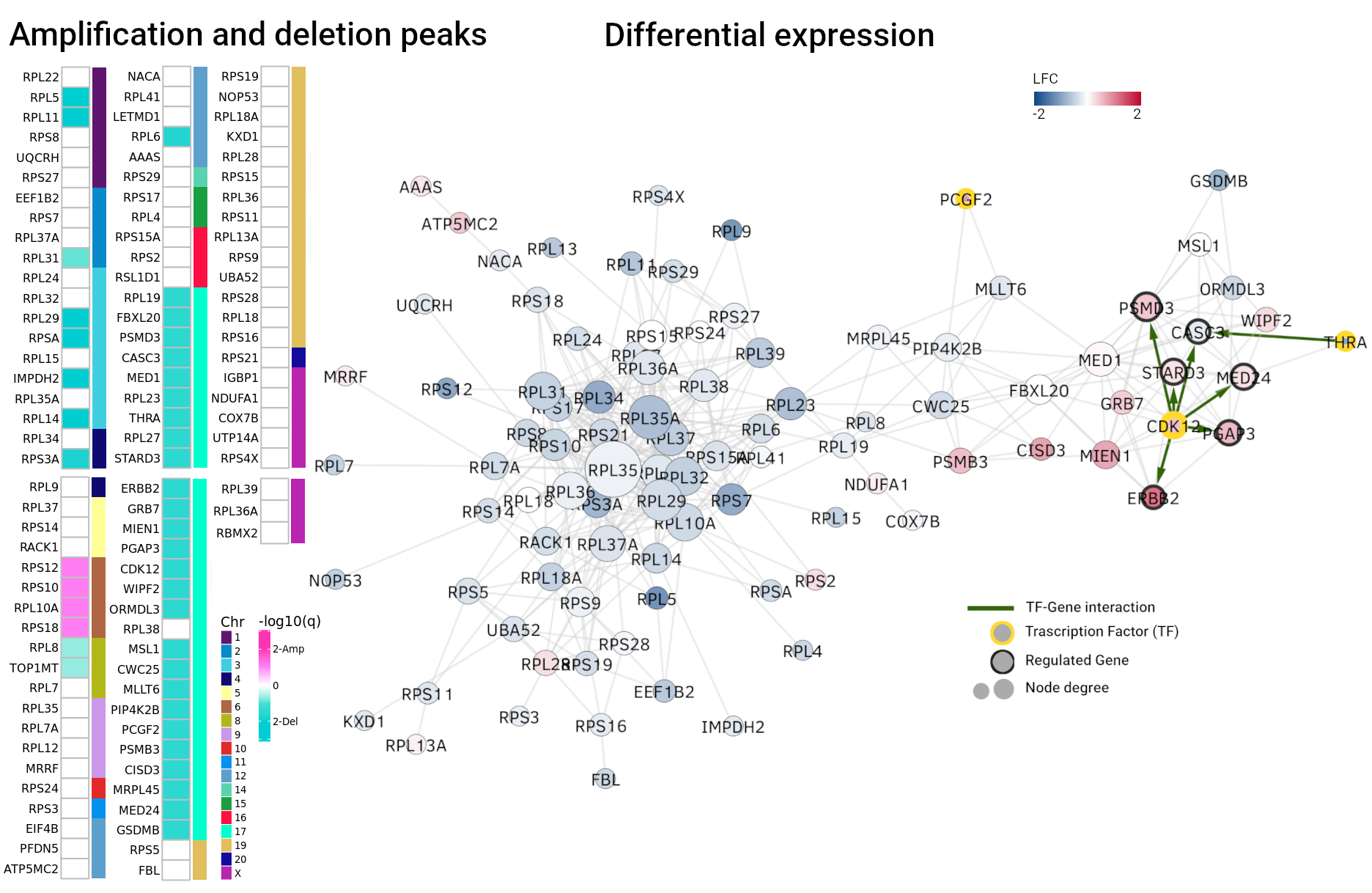

Supplement: Supplementary Figure 2 — RPL35 community. Left panel presents amplification and deletion peaks identified by GISTIC2, through pink and turquoise squares. Genes are ordered according to their corresponding chromosome. Right panel displays differential expression and regulatory interactions in genes in the community. [file Image_2.PNG]

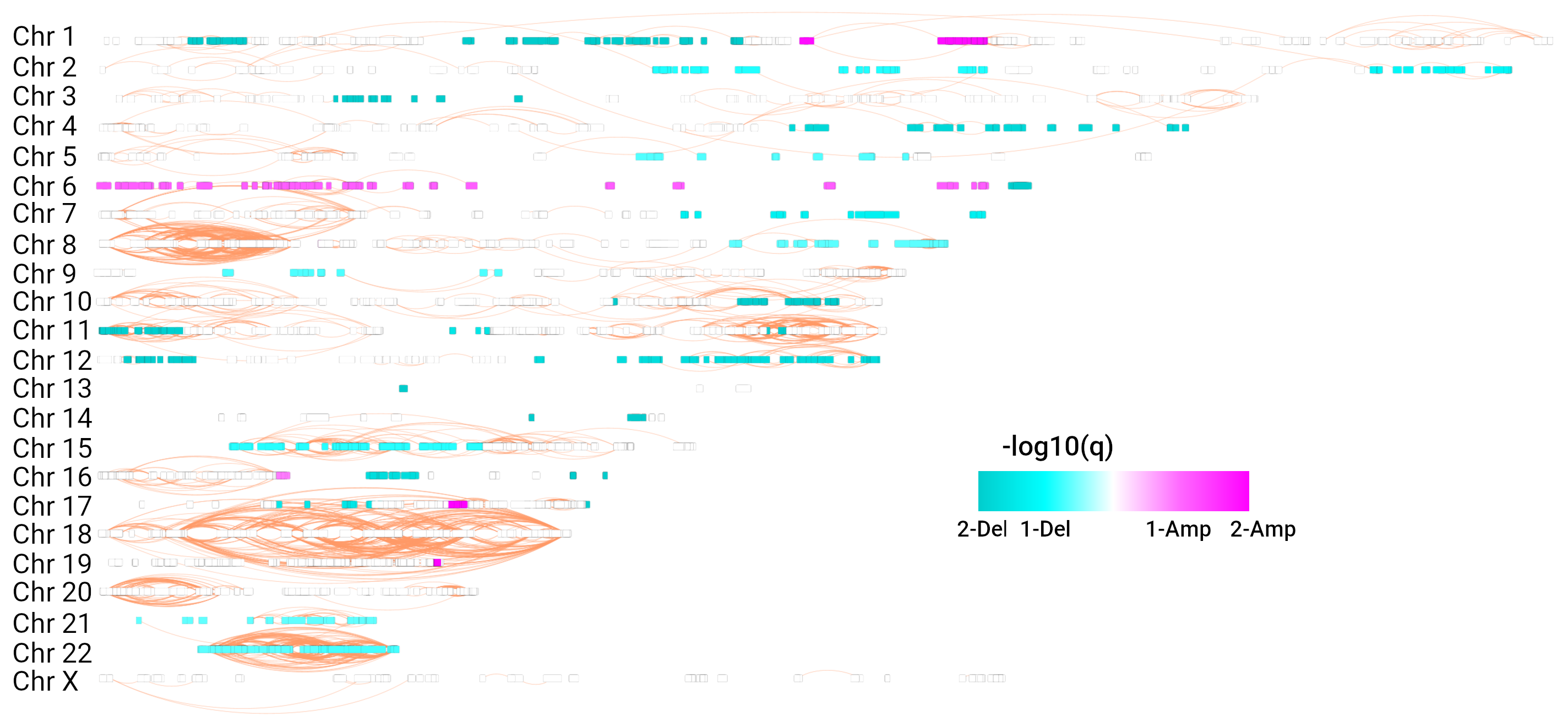

Supplement: Supplementary Figure 3 — Amplification and deletion peaks in cis- communities. Entire set of copy number alterations identified in intra-chromosomal communities. Genes are displayed according to their starting site. [file Image_3.PNG]

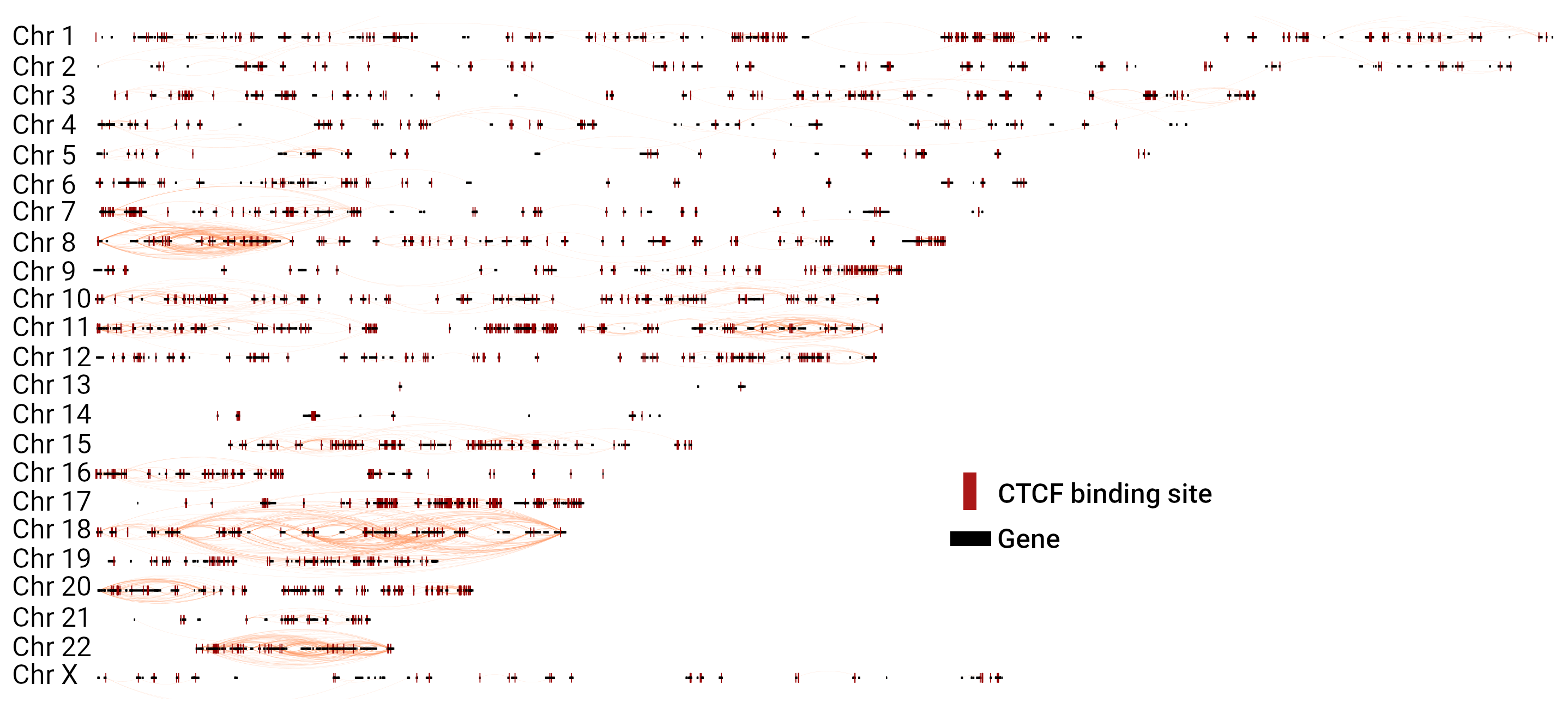

Supplement: Supplementary Figure 4 — CTCF binding sites distribution over cis- communities. Biding sites at a distance of no more than 50,000 base pairs from a gene in the community are displayed. [file Image_4.PNG]
